# Bacteria modulate microalgal aging physiology through the induction of extracellular vesicle production to remove harmful metabolites

**DOI:** 10.1038/s41564-024-01746-2

**Published:** 2024-08-14

**Authors:** Yun Deng, Ruyi Yu, Veit Grabe, Thomas Sommermann, Markus Werner, Marine Vallet, Christian Zerfaß, Oliver Werz, Georg Pohnert

**Affiliations:** 1 Institute for Inorganic and Analytical Chemistry, Friedrich Schiller University Jena, Jena, Germany; 2 Balance of the Microverse Cluster of Excellence, Friedrich Schiller University Jena, Jena, Germany; 3 Imaging Platform, Max Planck Institute for Chemical Ecology, Jena, Germany; 4 Department of Infection Immunology, Leibniz Institute for Natural Product Research and Infection Biology, Jena, Germany; 5 Department for Pharmaceutical/Medicinal Chemistry, Institute of Pharmacy, Friedrich Schiller University Jena, Jena, Germany; 6 Max Planck Fellow Group Plankton Community Interaction, Max Planck Institute for Chemical Ecology, Jena, Germany

**Keywords:** Microbial ecology, Metabolomics, Chemical ecology

## Abstract

The bloom and bust patterns of microalgae in aquatic systems contribute massively to global biogeochemical cycles. The decline of algal blooms is mainly caused by nutrient limitation resulting in cell death, the arrest of cell division and the aging of surviving cells. Nutrient intake can re-initiate proliferation, but the processes involved are poorly understood. Here we characterize how the bloom-forming diatom *Coscinodiscus radiatus* recovers from starvation after nutrient influx. Rejuvenation is mediated by extracellular vesicles that shuttle reactive oxygen species, oxylipins and other harmful metabolites out of the old cells, thereby re-enabling their proliferation. By administering nutrient pulses to aged cells and metabolomic monitoring of the response, we show that regulated pathways are centred around the methionine cycle in *C. radiatus*. Co-incubation experiments show that bacteria mediate aging processes and trigger vesicle production using chemical signalling. This work opens new perspectives on cellular aging and rejuvenation in complex microbial communities.

## Abstract (web summary)

The release of vesicles that shuttle harmful metabolites out of aging cells of the bloom-forming *Coscinodiscus radiatus* is modulated by bacteria.

## Main

Photosynthetic marine microalgae forming the phytoplankton carry out nearly half of global photosynthesis^1^. Plankton composition is highly complex with hundreds of co-existing species at a given time. Dependent on abiotic factors and biotic interactions, species composition varies dynamically, resulting in annual phytoplankton succession. Under favourable conditions, microalgae form blooms due to a more rapid proliferation of cells compared with their cell death. Blooming can result in the depletion of nutrients and consequently, cells are stressed and the population declines. Algal blooms thus develop from dormancy, proceeding to bloom initiation and persistence, then to a demise phase^2^. In certain situations, old blooms in their demise phase can be revitalized through the influx of nutrients, with blooming populations re-emerging^3^. Especially horizontal or vertical advection can restore favourable conditions leading to a revival of the bloom^4^. Thus, for example, enhanced phytoplankton productivity can be caused by flow disturbance and nutrient enrichment around an island^5,6^. Also, fluxes in ocean eddies lead to biological patchiness in the bloom, with nutrients seeding a replete community^7^. Refreshing of blooms with nutrients is also caused by upwelling of silicate-rich water. Diatoms can thrive by utilizing silicate brought to the surface by unstable ocean fronts^8^. Such situations can be experimentally triggered at a large scale in ocean fertilization experiments, where a limiting resource, such as iron, is added into the water and triggers proliferation of otherwise limited cells^9^. Common to all these phenomena is the rapid recovery of nutrient-limited cells from their stressed form and initiation of a new bloom. Despite the frequency of such events, the physiological processes that transform an ‘old’ and limited phytoplankton cell into a seeding ‘young’ population are not yet understood.

Microalgae can adapt to changing environments in multiple ways. Old and young cells differ substantially in their physiology, their metabolome and the secretion patterns of organic metabolites into the surrounding seawater^10–12^. Secretion processes that release dissolved organic material into the water can influence the entire plankton community by providing organic carbon for heterotrophs and mixotrophs^13^. Further, released chemical mediators can suppress the growth of competitors and modulate the performance of associated bacteria and grazers^14^. Release of organic material can also occur in the form of vesicles that shuttle diverse compounds and enzymes, thereby supporting the growth of heterotrophic bacteria or facilitating viral infection^15–17^.

The bacterioplankton community commonly found in association with algae also modulates blooms. Bacteria can promote or inhibit the proliferation of the algae using chemical mediators^18^. For example, bacteria can provide essential vitamins to algae, receiving, in turn, organic nutrients^19^. Other bacteria can adopt an algicidal lifestyle, killing algae and growing on the released resources^18^. Even dynamic shifts between these strategies are documented, illustrating the complexity of the connections^20^. As a consequence of these dependencies, bacterial community composition is also influenced massively by different algal species^21^. Rejuvenation of a plankton bloom thus has to be considered in the context of the associated bacteria, which we do in this contribution. We address the physiological and metabolic adaptations that algal cells undergo during recovery from nutrient limitation and the influence of associated bacteria on this process.

## Results

### Aging in *Coscinodiscus radiatus* cultures

**Fig. 1:**
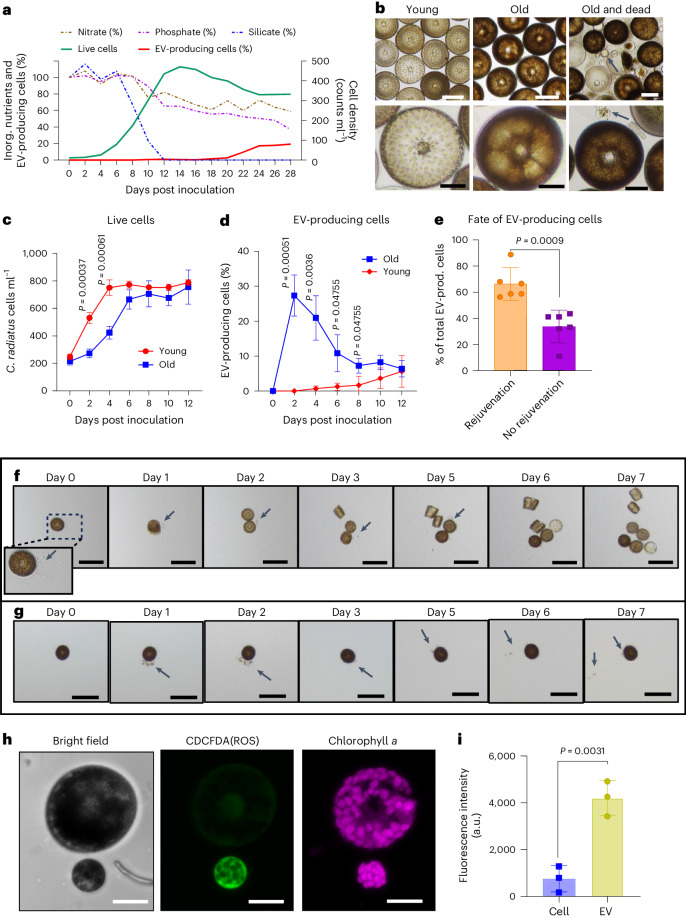
Aging physiology, EV production and rejuvenation in the diatom *C. radiatus.* **a**, The growth curve of *C. radiatus* along with concentrations of the inorganic nutrients nitrate, phosphate and silicate, and the proportion of EV-producing cells. Mean values from 6 biological replicates are plotted. **b**, Representative images of young (day 4–12), old (day 14–20) and dying (day >20) cells. Arrows indicate EVs. Scale bars, 200 µm (top row), 50 µm (bottom row). **c**,**d**, Growth (**c**) and proportion (**d**) of EV-producing cells of *C. radiatus* after inoculation of either young or old cells into fresh medium at day 0; *n* = 4 independent replicates, data presented as mean ± s.d. Multiple *t*-test was performed and statistical significance was determined using the Holm–Sidak method, with *α* = 0.05. **e**, Continuous tracking of single isolated old cells after inoculation into fresh medium for 1 week reveals that a higher percentage of cells that produce EV rejuvenate. Six single-cell tracking tests with a total of 335 cells from independent cultures were carried out. Mean ± s.d. of 6 replicates. Unpaired two-tailed *t*-test was employed for statistical analysis, *t* = 4.682, d.f. = 10. **f**,**g**, Two representative developments of rejuvenating (**f**) and non-rejuvenating (**g**) EV-producing cells. Arrows indicate EVs. Scale bar, 100 µm. Images are representative of 6 independent experiments. **h**, Image of a single EV-producing cell from cLSM three-dimensional (3D) scanning after CDCFDA staining. Average intensity of all *z*-axis stacks is given. Scale bar, 20 μm. Cells and vesicles were independently observed 3 times with consistent results. **i**, CDCFDA (ROS) signal intensity in the EVs and the producer cells after inoculation in fresh medium. A comparison of summed mean intensities of the *z*-axis stacks of the cells and their EVs is given. Three independent cells and associated EVs were analysed. Data are presented as mean ± s.d. of triplicates. Unpaired two-tailed *t*-test was performed, *t* = 6.393, d.f. = 4. No signal could be detected from non-stained cells (Supplementary Fig. 1). Source data

**Extended Data Fig. 1:**
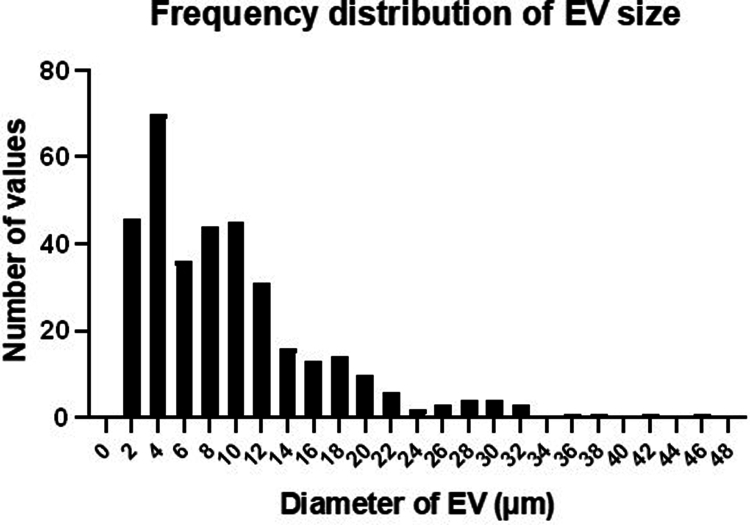
Distribution of EV size. All EVs visible in the field of vision of a microscopic picture were determined. The diameter of EVs was determined using the software imageJ. The sample was taken from an old culture with obvious EV production which was transferred into a 96 well plate after overnight inoculation. Source data

To monitor effects of aging and rejuvenation in microalgae, we established the diatom *Coscinodiscus radiatus* and its associated bacteria as a study system^22^. In nature, this alga is prominent in phytoplankton blooms and limited by the availability of inorganic nutrients^23^. In culture, rapid growth was observed as long as inorganic nutrients were available. We defined cells in this state as ‘young’. Under the culture conditions, silicate, required to build the biomineralized cell wall of *C. radiatus*, became limiting and cells stopped dividing (here defined as ‘old’ cells; Fig. 1a,b after day 14). Silicate, but not nitrate and phosphate, was depleted at this stage (Fig. 1a). Very old, dying and dead cells were abundant after day 20. At this time, the pronounced formation of extracellular vesicles (EV) was also evident (Fig. 1a). EVs have a wide size distribution spanning from 2 to 46 µm, with the majority of vesicles ranging between 2 and 12 µm (Extended Data Fig. 1).

**Extended Data Fig. 2:**
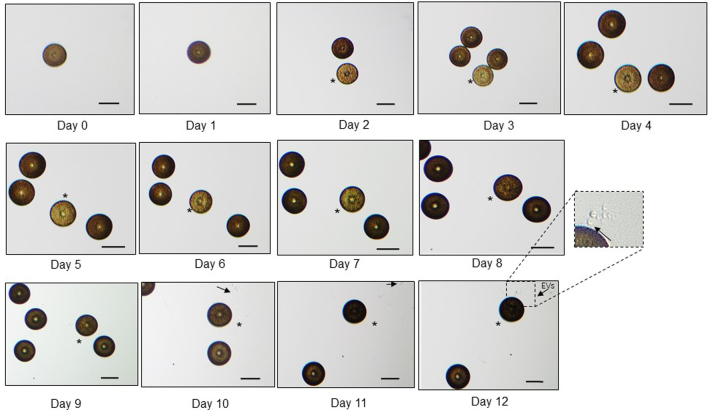
Tracking of single cells. Tracking of a single cell from young to old corresponding to the young inoculation in Fig. 1c and Fig. 1d. Tracking was performed by manually following a time-lapse picture series. The asterisk indicates the tracked cell. Arrows indicate EVs, scale bar = 100 µm.

During the exponential phase, most cells are dividing and have a homogeneous light pigmentation (Fig. 1b). In the late stationary phase, pigmentation of the cells becomes more intense but also variable (Fig. 1b), which indicates cellular aging of the microalgae^24^. To further inspect single cells, we switched from bulk cultures to a high-throughput small-volume cultivation in 96-well plates where the change in pigmentation and production rates of EV can be observed in single cells. Consistently, young cells inoculated into fresh medium divide and become more pigmented over time before starting to generate EVs (Extended Data Fig. 2).

Young cells with reduced associated bacteria (see Methods) from exponential or early stationary phase inoculated into fresh medium recovered faster than inoculations of old cells (Fig. 1c). The pronounced formation of EVs by the old cells during the recovery phase was striking (Fig. 1d and Supplementary Video 1). In young cells, the proportion of EV-producing cells was below 5%, while more than 25% of recovering old cells produced EVs (Fig. 1d). Single-cell tracking of six independent old cultures allowed determination of the fate of these EV-producing cells. EV production proceeds with increased rejuvenation (Fig. 1e–g), suggesting that EVs are involved in aging regulation. No evidence for a role of EVs in programmed cell death mechanisms was observed under these conditions (Supplementary Fig. 2). EV-producing cells that did not rejuvenate survived during the observation period of 7 days, confirming that EV production is not a result of programmed cell death. EVs are stable over several days; we did not observe the uptake of a vesicle by the producing alga or a neighbouring cell. The vesicles thus do not serve as a shuttle of metabolites between algal cells (see Supplementary Video 1).

Aging processes often occur along with accumulation of reactive oxygen species (ROS) that lead to cellular damage. Therefore, we tested for ROS by staining with the ROS-sensitive fluorescent probe CDCFDA (5(6)-carboxy-2′,7′-dichlorofluorescein diacetate). Distinct staining in EVs was observed (Fig. 1h). Observation of single diatom cells and their associated vesicles revealed that EVs have a significantly higher ROS content than the producing cells (Fig. 1h,i, and Supplementary Figs. 3 and 4), suggesting the active clearance of ROS by the cell.

### Bacteria modulate the aging of *C. radiatus*

**Fig. 2:**
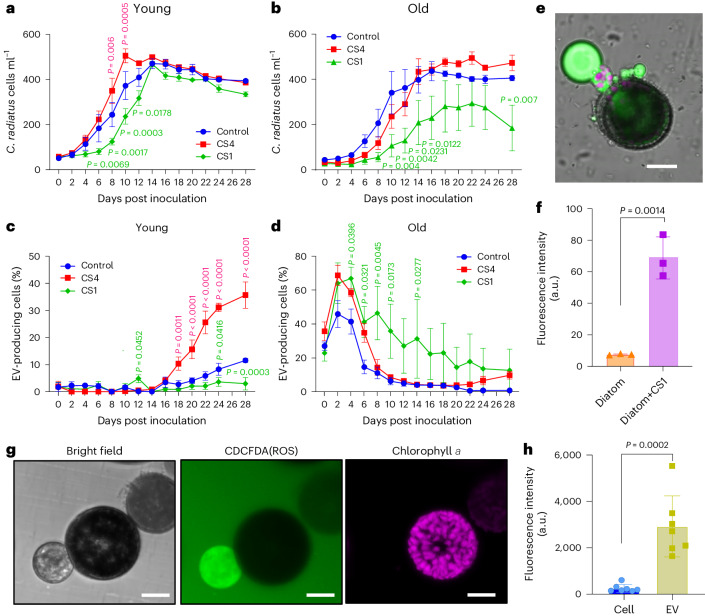
Bacteria modulate aging and EV production in *C. radiatus.* **a**,**b**, The effect of selected bacteria on the growth of *C. radiatus* was evaluated by co-inoculating young (**a**) or old (**b**) *C. radiatus* cells with the bacteria *Mameliella* sp. CS4 and *Marinobacter* sp. CS1, respectively. Purified *C. radiatus* served as control. **c**,**d**, The percentage of EV-producing algal cells. The red and green *P* values refer to significant differences between control and CS4, and control and CS1 treatments, respectively, within each time point. Data represent mean ± s.e. of 4 independent replicates within each time point. Dunnett’s test was applied for **a** and **b**, *α* = 0.05, and Fisher’s least significant difference (LSD) test was applied for **c** and **d**, *α* = 0.05. **e**, cLSM image of an old vesicle-producing *C. radiatus* cell after being exposed to CS1 (day 6 in **d**). Green fluorescence indicates ROS probe staining, pink fluorescence indicates chlorophyll *a*. Scale bar, 20 μm. **f**, Total ROS induced by the bacterium CS1 after 24 h incubation in a purified diatom culture compared with a CS1-treated culture (*n* = 3 independent replicates, mean ± s.e., unpaired two-tailed Student’s *t*-test, *t* = 7.923, d.f. = 4). **g**, Left: bright field microscopic image of an EV-producing cell and EV after treatment with bacterial CS1 filtrates (see Supplementary Fig. 1 for non-stained control). Middle: cLSM microscopic image of average CDCFDA fluorescence intensity of all *z*-axis stacks. Right: cLSM image of average chlorophyll a fluorescence intensity of all *z*-axis stacks. Scale bars, 20 μm. The experiment for image **g** was independently recorded in triplicate with consistent results. **h**, Comparison of mean fluorescence intensities of the *z*-axis stacks of the cell and EV (the region of interest was selected to exclude chloroplasts, as illustrated in Supplementary Fig. 6). Seven independent cells and associated EVs were analysed. Data are presented as mean ± s.d. Unpaired two-tailed *t-*test was performed, *n* = 7, *t* = 5.321, d.f. = 12. Source data

**Extended Data Fig. 3:**
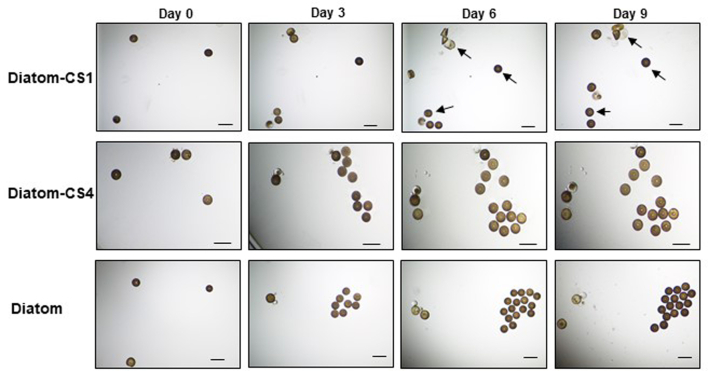
Tracking of old cells. Tracking of old *C. radiatus* cells in the presence (upper and middle) and absence (lower) of bacteria CS1 and CS4 from day 0 to day 9 post inoculation into fresh medium. Scale bar = 200 µm. Arrows indicate malformation and non-viable cells. The images are representative of three independent experiments.

In nature, algae are always associated with bacteria that influence their performance^2^. To determine how bacteria or bacterial metabolites influence the aging of algal cells, we performed a series of co-culture and metabolomics experiments. We selected *Mameliella* sp. CS4 and *Marinobacter* sp. CS1, two bacteria that co-occur in the natural environment of the alga^22^, for the treatments. Strikingly, young and old diatom cells respond differently to the presence of the bacteria (Fig. 2). Young *C. radiatus* reach faster higher cell counts in the presence of CS4 and are slowed in culture development by CS1. Diatoms in both bacterial treatments reached similar cell densities compared with the control after 14 days (Fig. 2a). In contrast, the growth of old diatoms was delayed by CS1 that also resulted in overall reduced cell counts (Fig. 2b). These bacteria also caused malformation and non-viable offspring (Extended Data Fig. 3). In treatments with CS4, cell counts of old diatoms reached those of controls with a slight delay (statistically non-significant) (Fig. 2b). As can be seen in Fig. 2d, both bacteria induce vesicles. During vesicle production, both bacteria delay growth. In the case of CS4, vesicle production ceases after 8 days when growth reaches values similar to those in the control. In the case of CS1, vesicle production and growth suppression persist for the entire experiments.

In young cells, EV production was only observed in late growth phases and was substantially promoted by CS4 (Fig. 2c). In strong contrast, more than 40% of all old diatom cells produced vesicles immediately after inoculation. CS1 and CS4 induced higher than 60% vesicle-producing cells. Vesicle production lasted over a longer period in CS1 compared with CS4 treatments, and vesicle production was significantly lower in controls (Fig. 2d). When the diatom cells were treated with a bacteria-free filtrate of a CS1 culture, EV production was induced in a dose-dependent manner (Supplementary Fig. 5). The increased vesicle production in CS1 treatments proceeded with elevated ROS concentrations in cells and vesicles (Fig. 2e,f). As described above, ROS were found in higher concentrations in EVs than in cells (Fig. 2g,h).

**Extended Data Fig. 4:**
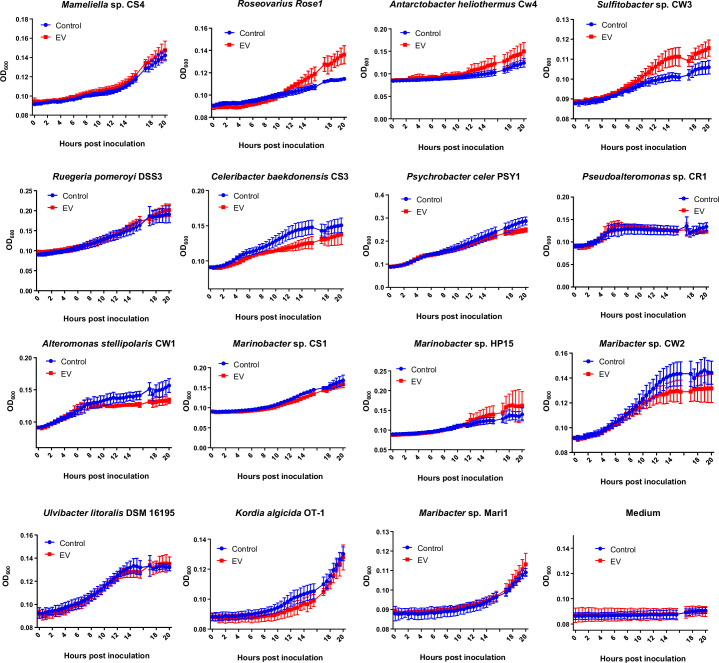
Effect of EV extracts on bacteria. Effect of the extracts of EVs on the growth of bacteria. Fifteen bacterial strains were selected from a previous survey of bacteria – algal interaction^22^ for the assay. The extract of EVs was added into each bacterial culture. The extract not containing EVs was set as control. A medium blank not containing bacteria is given. Data represent mean ± SD from independent triplicates. Source data

To evaluate whether the vesicles themselves influence the associated bacteria, we conducted a screening of 15 marine bacteria exposed to vesicle extracts. Out of the tested bacteria, two Roseobacteraceae were promoted in growth by vesicle extracts, while three Gamma-proteobacteria and another Roseobacteraceae were inhibited. Since inhibition was manifested during later growth phases, no immediate toxicity of the vesicle content can be concluded. All other tested strains remained unaffected (Extended Data Fig. 4).

**Fig. 3:**
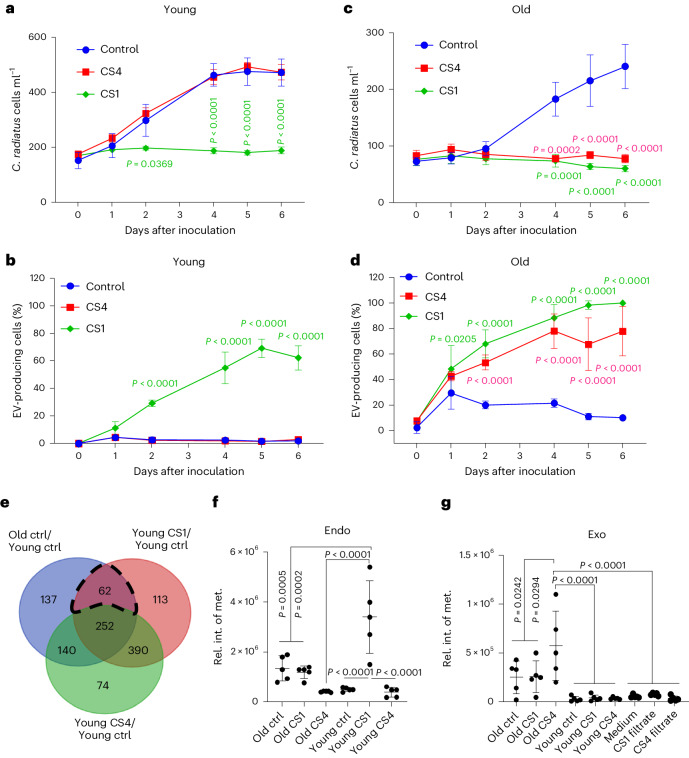
Interaction with bacteria inhibits growth, induces vesicle formation and upregulates methionine in *C. radiatus*. **a**–**d**, Effects of 0.2 µm filtrates of CS1 and CS4 on the growth and vesicle production of *C. radiatus* cultures seeded with young (**a** and **b**) and old (**c** and **d**) cells. The red and green *P* values refer to significant differences between CS4 and control, and between CS1 and control, respectively. Within the same time point, Dunnett’s test was used (*n* = 4 independent replicates, mean ± s.e.). The same incubation conditions were applied in the untargeted metabolomics experiment. **e**, Venn diagram analysis of the differentially expressed endometabolites among young CS1/young control, old control/young control and young CS4/young control (FC > 1.5, *P* < 0.05). The intersection (dashed area) of young CS1/young control and old control/young control contained 62 differentially expressed metabolites with 20 tentatively annotated metabolites. Out of these, methionine (met.) was found in higher abundance in endometabolites of old and CS1-treated diatoms. **f**, Ion counts of integrals in mass spectra. **g**, Methionine was also among the elevated exometabolites in all old cultures. Significance was assessed using one-way analysis of variance (ANOVA) with Tukey’s test. Data are presented as mean ± s.d. from 5 independent replicates. Source data

**Extended Data Fig. 5:**
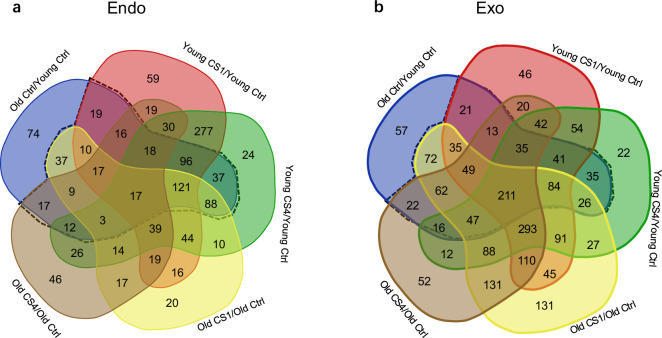
Comparisons of endo- and exometabolites. Pairwise comparisons of endo- (**a**) and exometabolites (**b**): old cultures control (Ctrl)/young cultures Ctrl; young cultures treated with CS1/young cultures Ctrl; young cultures treated with CS4/young cultures Ctrl, old cultures treated with CS1/old cultures Ctrl, and old cultures treated with CS4/old cultures Ctrl. The dashed lines highlight the groups of metabolites commonly regulated during aging as well as in response to bacterial treatments. Venn diagrams show the significant up- or downregulated (fold change FC > 1.5, p < 0.05) endometabolites. Source data

To explore the metabolic and physiological processes behind the responses of the diatom to bacteria, we conducted a metabolomic analysis and identified substantially regulated metabolites in pairwise comparisons (Extended Data Fig. 5). Young and old *C. radiatus* cells were therefore inoculated with bacteria or treated with sterile filtrates of the respective bacterial cultures and incubated for 3 days so that responses can be manifested. Effects of filtrates resulted in patterns similar to those of living cells (Fig. 2a–d), indicating a chemically mediated interaction. CS1 extracts inhibited the growth of young diatoms and induced EVs during the first days of inoculation, while CS4 extracts did not affect growth and EV production (Fig. 3a,b). Cultures seeded with old cells were suppressed in growth by both bacterial extracts throughout the experiment (Fig. 3c). Induction of vesicle formation was even higher with bacterial extracts than in the co-cultures (Supplementary Fig. 5). The stronger effects are probably due to the higher concentration of metabolites in the extracts than in control cultures. However, it should be noted that co-cultures and treatments with extracts do not necessarily compare 1:1. Co-culture experiments allow suppression and degradation of signal metabolites by the partner, but also induction of chemical mediators that would not be observed in extracts.

Differentially expressed metabolites (fold change (FC) > 1.5, *P* < 0.05) were identified in pairwise comparisons of the treatments using liquid chromatography–mass spectrometry (LC–MS) data from day 3 (Methods). Comparisons of endo- and exometabolites between old control/young control, old CS1/old control, old CS4/old control, young CS1/young control and young CS4/young control were plotted in Venn diagrams (Extended Data Fig. 5). Of the differentially expressed features found by the statistical analysis, 87.5% of endometabolites and 93.0% of exometabolites are regulated during aging as well as in response to bacterial treatments. Algal responses to aging and bacteria are thus partly shared.

Metabolomics analysis was used to identify candidate metabolites that could be responsible for the growth inhibition and EV induction by CS1 in *C. radiatus*. Therefore, endometabolites upregulated in the pairwise comparison of young CS1/young control, but not in the comparison of young CS4/young control were identified. This resulted in 175 features. To reduce this dataset, we searched for metabolites that are also upregulated in the comparison of old control/young control. This resulted in 62 features as candidates that might be connected to aging and EV production (Fig. 3e). In total, 20 of the 62 features could be annotated. Intracellular methionine was among the metabolites that were significantly upregulated in both the CS1 filtrate-treated young cells and old cells (Fig. 3f,g). CS1 inhibits the division of young cells (Fig. 3a). Therefore, methionine, which is used for protein biosynthesis during growth, could accumulate in the cells. In contrast, old cells can more efficiently export methionine out of the cell by EV production (Fig. 3g; note that metabolites contained within EVs are included in the exometabolome pool).

### Methionine cycle regulation in the aging physiology

**Fig. 4:**
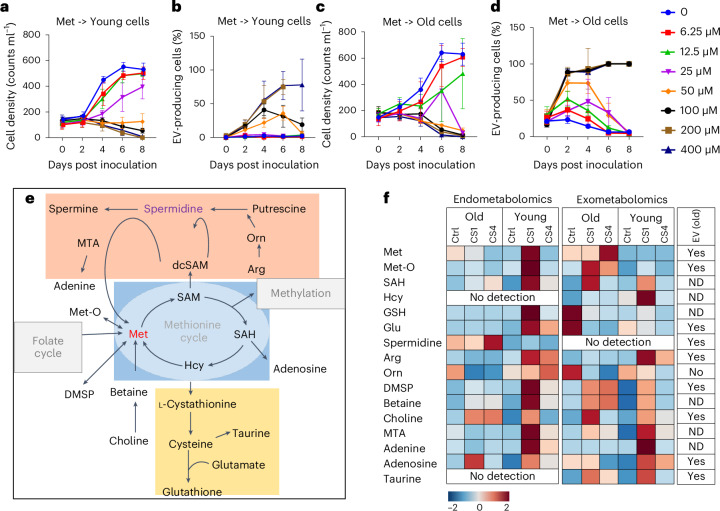
Regulation of the methionine cycle and related metabolites in *C. radiatus* and induction of aging physiology. **a**–**d**, Bioassays of the effects of methionine on growth (**a** and **c**) and EV production (**b** and **d**) of young and old *C. radiatus*. Data are presented as mean ± s.d. from *n* = 3 independent replicates. **e**, Methionine cycle and its coupled transsulfuration pathway and polyamine biosynthesis. **f**, Heat map showing the relative abundance of members of the methionine cycle and related metabolites in *C. radiatus* endo- and exometabolomes. Treatments include purified old and young cells without further supplements (Ctrl) or with bacterial filtrates from CS1 or CS4 cultures. Heat maps indicate the relative abundance after average centred normalization (see Extended Data Figs. 6 and 7 for the underlying data and statistical analysis). The column at the right side indicates whether the metabolites were detected in purified EVs (after FACS). Met-O, methionine sulfoxide; Hcy, homocysteine; SAM, *S*-adenosylmethionine; SAH, *S*-adenosyl homocysteine; dcSAM, decarboxylated *S*-adenosylmethionine; GSH, glutathione; Orn, ornithine; MTA, methylthioadenosine. All listed metabolites besides MTA were identified by comparison with authentic standards. Source data

Encouraged by the consistent regulation of methionine, we conducted bioassays with this amino acid. Methionine inhibited growth (IC_50_ = 36.9 µM for young and 15.6 µM for old cells, IC_50_ refers to the inhibitory concentration where 50% of the growth is inhibited) and induced EV production (EC_50_ = 63.2 μM for young and 32.1 μM for old cells) (Fig. 4a–d). A 14 h time-lapse video after treatment with 50 μM methionine confirmed the higher EV production in old compared with young cells (Supplementary Video 2).

Given the regulation and activity of methionine, we mined our mass spectrometry data for other members of the methionine cycle and coupled pathways, including betaine metabolism, polyamine synthesis, dimethylsulfoniopropionate (DMSP) biosynthesis and transsulfuration pathways (Fig. 4e). Along with the pronounced upregulation of methionine in the treatment of young algae with the bacterium CS1, methylthioadenosine, adenine, methionine sulfoxide, DMSP, betaine, ornithine, arginine, adenosine, homocysteine, glutathione, glutamate and taurine were upregulated. Most of these metabolites were also elevated in the exometabolome of old diatoms (Fig. 4f).

Of these upregulated metabolites, the methionine cycle metabolites methionine, homocysteine and methionine sulfoxide were selected for bioassays on growth inhibition and induction of vesicle formation. Also reduced glutathione, cysteine, spermidine and arginine were chosen due to their activity in algal physiology and chemical ecology. Quantification of methionine revealed concentrations of 1–4 µM in the exometabolome of young cells and 10–40 µM in the exometabolome of old cells. Concentrations in bioassays were selected to cover this range. The IC_50_ and EC_50_ (concentration of a metabolite that induces half-maximal proportion of EV producing cells) values are given in Supplementary Table 1 and are documented in the Extended Data Fig. 8. Except for methionine sulfoxide, none of the tested metabolites inhibited growth comparable to or stronger than methionine itself. Homocysteine, glutathione and cysteine were only active at elevated concentrations, while the other tested metabolites did not affect growth or EV production. To evaluate the possible involvement of methionine in methyl transfer, we also tested methyl methionine, which exhibited the strongest EV induction and growth inhibition activity (Extended Data Fig. 8). In general, old cells are more sensitive to the treatments.

Polyamine (PA) synthesis is another pathway related to the methionine cycle, as decarboxylated *S*-adenosylmethionine (dcSAM) provides aminopropyl groups for the synthesis of spermidine, a putrescine precursor. PAs are metabolites involved in the regulation of aging processes^25^. From the metabolomics data, spermidine content was only slightly elevated in old cells and did not promote growth inhibition or EV production. In the presence of PAs, CS1 significantly inhibited growth and promoted EV production in young *C. radiatus* (Extended Data Fig. 9).

Betaine and folate are methyl donors for methionine synthesis^26^. When we supplemented 0.2 mM betaine to cultures of old cells in the presence of CS1, growth was restored and EV production was reduced significantly. Folate also reduced EV production (Extended Data Fig. 9).

### EV production as a means to eliminate detrimental metabolites

To verify whether vesicle production is a means to export metabolites that are detrimental to the cellular performance of *C. radiatus*, we investigated the metabolic profile of the vesicles. Therefore, we developed a fluorescence-activated cell sorting (FACS) protocol to purify vesicles after ROS fluorescent probe labelling (Extended Data Fig. 10a). In the FACS analysis, we identified five regions that contain medium-sized vesicles, large vesicles, small vesicles, chloroplasts and high-granularity vesicles according to size, granularity, ROS content and chlorophyll *a* fluorescence. We selected large, stained EVs for sorting (Extended Data Fig. 10b–f). Methionine and other metabolites connected to the methionine cycle including DMSP, choline, glutamate, methionine sulfoxide and adenosine were detected in purified EVs (Fig. 4f and Extended Data Fig. 10g).

**Fig. 5:**
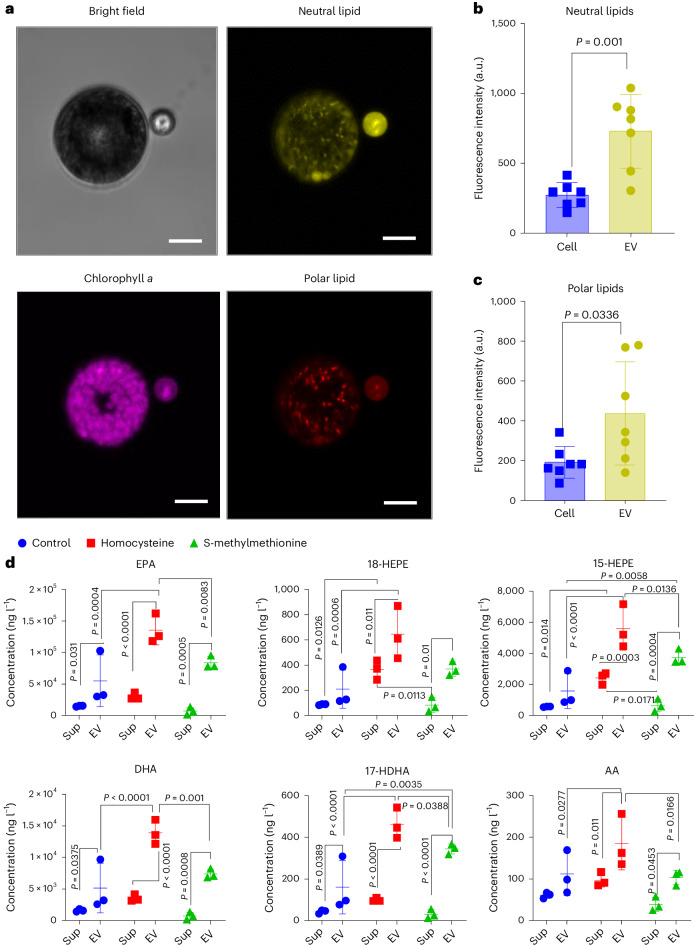
Microalgae excrete fatty acids and oxylipins by EV production. Quantification of Nile Red (neutral lipids in yellow and polar lipids in orange) signal intensity in the EV and the producer cell after inoculating in fresh medium. **a**, Image of an EV-producing cell from cLSM 3D scanning. Average intensity of all *z*-axis stacks is presented. No signal was detected from non-staining cells (Supplementary Fig. 7). Scale bars, 20 μm. **b**,**c**, Comparison of maximal intensities of neutral lipids (**b**) and polar lipids (**c**) of 7 independent pairs of cells and associated EVs. Data represent mean ± s.d. Unpaired two-tailed *t*-test for neutral lipids, *t* = 4.340, d.f. = 12; for polar lipids, *t* = 2.398, d.f. = 12. **d**, Quantification of free fatty acids and oxylipins in the EV-enriched fraction and the supernatant (*n* = 3 independent replicates). EPA, eicosapentaenoic acid; HEPE, hydroxyeicosapentaenoic acid; DHA, docosahexaenoic acid; HDHA, hydroxydocosahexaenoic acid; AA, arachidonic acid. Data are presented as mean ± s.d. from triplicates. Fisher’s LSD test was performed. Source data

Abundant lipids and related metabolites, such as acetyl-l-carnitine, butyrylcarnitine and eicosapentaenoic acid (EPA) were elevated in old cells and FACS-purified EVs. Confocal laser scanning microscopy (cLSM) observation after staining with the lipid probe Nile Red indicated lipid droplets in EVs (Fig. 5a). Both neutral lipids and polar lipids have significantly higher concentrations in EVs than in the producer cells (Fig. 5b,c). A targeted analysis of fatty acids and fatty acid oxidation products (oxylipins) known to regulate diatom metabolism and defence was conducted (Fig. 5d)^27^. To collect EVs, old cells were stimulated for EV production by inoculation into fresh medium for 2 days. In parallel, the old cells were treated with 100 μM homocysteine or 10 μM *S*-methyl methionine to enhance EV production. EPA and the EPA-derived oxidized fatty acids (oxylipins) 18-hydroxyeicosapentaenoic acid (HEPE), 15-HEPE, 12-HEPE, 11-HEPE and 5-HEPE were dominant in the EVs but were less in the supernatant or in cells. Also, docosahexaenoic acid (DHA) and the DHA-derived lipids 17-hydroxydocosahexaenoic acid (HDHA), 14-HDHA, 13-HDHA, 10-HDHA, 7-HDHA and 4-HDHA followed this pattern. Less arachidonic acid (AA) and AA-derived oxylipins were detected (Fig. 5d). EV production is thus a means for the microalga to excrete not only ROS and methionine cycle-related metabolites but also fatty acids and oxylipins.

## Discussion

The aging of microalgae under nutrient-limiting conditions and rejuvenation after nutrient influx is one of the determinants of productivity in phytoplankton. The high turnover of plankton contributes massively to global carbon fixation and element cycling. Processes that influence the population dynamics thus control the marine food web and consequently, also climate functioning. To get a deep insight into the aging physiology and rejuvenation of phytoplankton, we investigated *C. radiatus*, a widely occurring bloom-forming diatom. We set up cultures that became limited in silicate, an essential nutrient required for the formation of the biomineralized diatom cell walls. Silicate can become limiting during diatom blooms under natural conditions, as, for example, during spring blooms in Arctic and temperate waters^28,29^. Due to upwelling, nutrient influx from rivers or mixing through currents, nutrient-limited populations can be transferred to nutrient-replete conditions and start division again. We mimicked such events by exchange of the growth medium in laboratory cultures.

During the development of the cultures from nutrient-replete fast-dividing stages to limited aged cells, we observed changes in pigmentation and metabolism. These changes are consistent with results from previous microscopic and metabolomic surveys of diatoms^10,30^. Changes in pigmentation can occur with altered photosynthetic activity as observed in the microalga *Chlamydomonas reinhardtii*^31^.

Young, that is, exponentially dividing, and old, nutrient-limited non-dividing cells also differed in their growth patterns when transferred to nutrient-replete medium. The lag phase of the young cells was significantly shorter than that of old ones. It is widely accepted that aging cells accumulate damaged cell constituents that are harmful to division or recovery^32^. According to our findings, this is also true for aged diatoms.

**Fig. 6:**
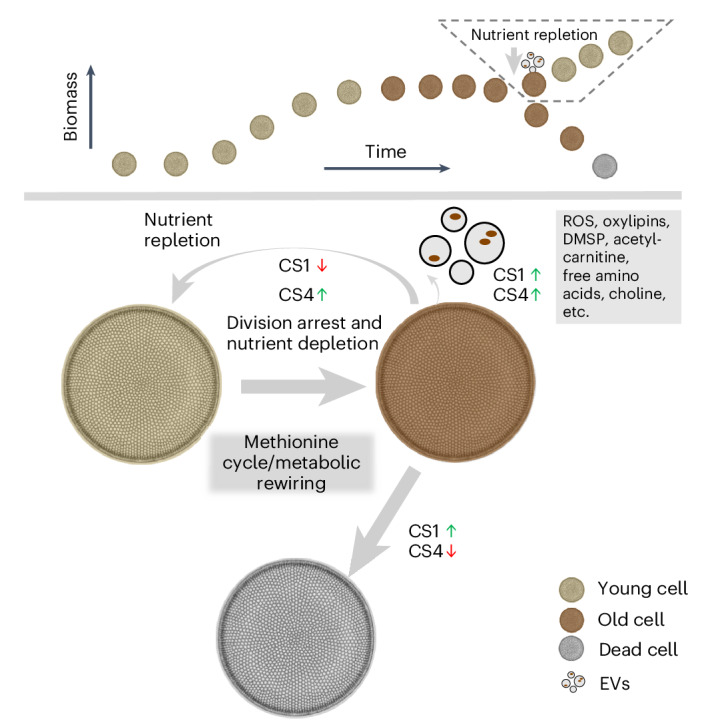
A model graph describing how bacteria modulate aging physiology in microalgae via the methionine cycle and induced EV production. Top: diagram indicating that physiological aging of the diatom occurs during late stationary phase according to the growth curve. Old cells become more pigmented and release EVs during the late growth stage when mortality is already high. Upon influx of nutrients, EV production and reduction of pigmentation occur with cell proliferation. Bottom: diagram showing how old diatom cells rejuvenate under the production of EVs that shuttle out detrimental metabolites. The process is regulated by the methionine cycle and related metabolites. The influence of the bacteria CS1 and CS4 on the processes is represented by arrows that refer to suppression and promotion, respectively.

To rejuvenate, cells have to eliminate detrimental constituents by enzymatic degradation or excretion^33^. We observed increased secretion of vesicles in old cells that were transferred to fresh medium. Nearly 30% of the cells initiated vesicle production. The process lasted until the cultures seeded with old cells reached cell densities similar to those seeded with the young cells. Vesicle production and recovery of fitness are thus synchronous in bacteria-free cultures, but synchronicity can be broken by bacteria. Vesicles are rich in ROS that cause cellular damage^34^ and also contain oxylipins—metabolites often associated with diatom stress metabolism and defence^27,35^. Our results suggest that EVs serve as a damage disposal mechanism. The shuttling of oxylipins out of the cells might also facilitate chemical defence by targeting lipophilic vesicles against pathogenic bacteria^27^. Taken together, vesicles shuttle harmful ROS and oxylipins but also fatty acids out of the aged cells and might provide a key for rejuvenation (Fig. 6).

The use of vesicles to excrete harmful metabolites is also a strategy known from bacteria that produce so-called minicells that lack chromosomes, cannot proliferate, and die^36^. Bacteria are rejuvenated by the disposal of the minicells and their daughters divide faster, very similar to the observations made here for diatoms. In the phytoplanktonic coccolithophore *Emiliania huxleyi*, virus infection occurs with vesicle production, with vesicles acting as a proviral signalling mechanism^16^.

In our set-up, spontaneous vesicle production was also observed in old cultures containing high proportions of dead cells. Given the chemical content of the vesicles, this mechanism might be the ultima ratio to prolong survival under highly adverse conditions. Sheldrake proposed in his rejuvenation hypothesis that damaged cell constituents build up in all cells, but cells can be rejuvenated either by growth and cell division or by excreting damaged cell constituents^32^. Since division is not an option under nutrient-limited conditions, the way out for old diatoms could thus be the observed EV excretion.

Since algae in nature do not occur in axenic states but rather in communities associated with bacteria, we tested how the two bacterial strains CS1 and CS4 found associated with *C. radiatus* blooms influence rejuvenation. While CS1 delayed the regrowth of algae after nutrient intake, CS4 did not cause this effect. Despite this difference, both bacteria induced a more pronounced vesicle formation compared with the purified control within the first days after nutrient intake. In CS1 treatments, vesicle formation lasted throughout the regrowth and prevented full recovery. These processes are mediated by chemical signals since the application of spent medium from the bacterial cultures triggered similar responses. It is well known that bacteria can modulate algal growth and metabolism to obtain resources for their heterotrophic growth. Thus, for example, the growth promotion of the coccolithophore *E. huxleyi* is mediated by tropodithietic acid released by the α-proteobacterium *Phaeobacter gallaeciensis*^20^. We previously demonstrated that algal growth is modulated by the bacteria CS1 and CS4 as well^22^. Here we relate this growth modulation to chemical signalling and, in addition, to the induced vesicle formation. The content of organic material including fatty acids and amino acids suggests that EV can provide substrates to the bacteria. However, under our experimental conditions, we found that only 2 out of 15 tested bacteria grew better in the presence of EV extracts. This result, however, might not translate directly into an ecological scenario since alternative carbon sources were present in the test medium that are not found in the very dilute environment of the plankton. The two different strategies pursued by bacteria might serve the same purpose. Inducing vesicles and restoration of growth might be considered as a strategy to initially provide large quantities of organic material to interacting bacteria before sustained release of metabolites by a healthy culture. However, the induction of EVs over the entire growth period could also result in the provision of nutrients.

To unravel the metabolic processes associated with rejuvenation in the presence or absence of bacteria, we selected a comparative metabolomics approach. Therefore, the intracellular metabolites (endometabolome) relevant to understanding physiological responses and the extracellular (exo) metabolites relevant for communication were monitored. In accordance with the literature, aging occurs with a remodelling of the endo- and exometabolome^10,37^. In the presence of bacteria, additional metabolomic changes were observed. CS1 that induced vesicle production and inhibited proliferation also had a massive impact on the metabolism of both young and old cells. Methionine was consistently upregulated in old cultures as well as in the CS1 treatments. The bacterium also induced increased concentrations of glutathione, betaine and biosynthetically related metabolites, indicative of a disbalance due to senescence. Especially the release of methionine into the exometabolome of old cells was pronounced. The regulation of the methionine cycle, betaine metabolism, polyamine synthesis, oxylipin production and methylation that we found in diatoms is also key to the aging physiology of humans^38^, animal models^39^ and plants^40^.

We tested the effect of methionine cycle-related regulated compounds on the proliferation and vesicle production of the algae. Methionine inhibits growth and induces vesicle production as did *S*-methyl methionine. Since methionine and related compounds are also detected in EVs, their production might represent a controlled mechanism to release these harmful metabolites in addition to ROS, fatty acids, oxylipins, free amino acids, choline and the osmolyte DMSP. This further supports the notion that vesicles might act as shuttles for metabolites that accumulate during cellular aging. Such a mechanism is consistent with the view that cells can be ‘immortalized’ by excreting harmful constituents in extracellular vesicles^32^.

## Conclusion

We characterize the aging physiology of a diatom which occurs with a metabolic reorganization. Rejuvenation upon nutrient intake is associated with the production of vesicles that shuttle adverse metabolites out of the old cells. Bacteria significantly influence the aging process by regulating the methionine cycle and by the induction of EV production. Our study indicates that the control of aging in diatoms shares conserved mechanisms observed in bacteria, higher plants, animals and humans. On a global scale, our findings may provide new perspectives for understanding and modelling phytoplankton ecological succession.

## Methods

### Strains, media and cultivation

The diatom *C. radiatus* was obtained from the Roscoff culture collection RCC 7277 (https://roscoff-culture-collection.org/rcc-strain-details/7277). The bacteria *Mameliella* sp. CS4 and *Marinobacter* sp. CS1 were isolated in our lab previously^22^. *C. radiatus* was cultivated in sterile Guillard’s (f/2) enrichment medium (G9903, Sigma-Aldrich) prepared with natural seawater (ATI). To optimize the growth of diatoms, silicate was added to reach the final concentration of 400 μM and the medium was noted as f/2+Si. Depending on the volume, cultures were maintained in tissue culture flasks (T175 for 150 ml cultures, T25 for 20 ml cultures, Sarstedt). Cultures (300 μl) were grown in 48-well plates, and 100 μl cultures were grown in 96-well plates (Sarstedt). Cultures were maintained at 13 °C with a light:dark cycle of 15 h:9 h and light intensity of 25 μmol m^−2^ s^−1^ for running experiments and 20 μmol m^−2^ s^−1^ for storage.

For the preparation of purified cultures, we applied a previously used method to minimize the bacterial community from *C. radiatus*^22^. Briefly, the cells were washed with 20 μg ml^−1^ Triton X-100 (X100, Sigma-Aldrich) on a filtering device (43-50040-51, 40 μm pore size, pluriSelect), followed by treatment with the antibiotics kanamycin (50 μg ml^−1^) and ciprofloxacin (20 μg ml^−1^) (K1377 and 23265, Sigma-Aldrich) overnight. After another filtering process, the antibiotics were removed by repeated centrifugation and washing with excess fresh f/2+Si medium. Cells were resuspended in a sterile Falcon tube. Young cells were stored under low light intensity in an incubator and used within 1 week. Old cells were used for the experiment on the same day of the filtration process. No bacteria were detectable under the microscope and after plating out on marine broth (MB, Sigma-Aldrich) agar plates (1.5% agar). Of note, since the diatoms are closely associated with bacteria, the cultures were strongly reduced in bacteria after the treatment but not necessarily entirely axenic.

Bacterial cultures were prepared from single colonies picked from marine broth (MB 76448, Sigma-Aldrich) agar plates (1.5% agar). Picked colonies were transferred into 3 ml liquid MB medium in 10 ml sterile tubes and grown at 28 °C with 150 r.p.m. shaking. When the cultures reached the late exponential phase at an optical density at 600 nm (OD_600_) of 0.4–0.7, 30 μl were transferred into 3 ml of fresh MB. When the cultures reached an OD_600_ = 0.4–0.7, they were collected by centrifugation at room temperature, washed two times with f/2+Si medium to remove the MB medium and resuspended in f/2+Si medium to reach an OD_600_ = 0.4 (~5 × 10^6^ cells per ml).

### Inorganic nutrients

For the determination of the inorganic nutrient content, *C. radiatus* RCC 7277 cultures were grown in 150 ml of f/2+Si medium as described above. Every other day, 2 ml were sampled and 100–300 μl of this sample were transferred to 96-well plates for counting of live and EV-producing cells. Nitrate, phosphate and silicate were determined by spectrophotometric methods following previously reported procedures^41^.

### *C. radiatus* aging

Young and old cultures of *C. radiatus* were selected on the basis of two criteria: (1) The cultivation time after initial inoculation was less than 14 days for young cultures and longer than 14 days but less than 20 days for old cultures grown in 150 ml medium. (2) When most of the cells in the culture showed light yellow pigmentation under the microscope and the chloroplast spots were visible with a clear edge, the culture was regarded as young. When most of the cells in the culture showed dark brown pigmentation, the culture was classified as old (Fig. 1b).

### Cell counting, time-lapse video capture and vesicle observation

Algal cell counting was done under a Leica DM2500 microscope with a scanning objective lens (×4) combined with a ×10 eyepiece lens. The microscope was equipped with a CCD system and the software NIC-elements D 4.30.00 was used for the photographic documentation. Live and dead cells were distinguished according to previous criteria^22^ and live cells were counted to monitor algal growth. When the individual live cell is attached to or surrounded by vesicle(s), this cell was recorded as an EV-producing cell. The number of EV-producing cells was normalized to the number of total live cells to give the EV-producing cell percentage. For 150 ml cultures, samples of 300 μl were pipetted to 96-well plates. After inoculation of the well plate at 13 °C for 6 h to allow time for EV production, the live cells and EV-producing cells were recorded as described above. For the alga cultivated in well plates, we directly counted the total cells from each well (100 μl for 96-well plate and 300 μl for 48-well plate). The counting was done within 1 h in the microscope room where the temperature was maintained at 18 °C. No obvious negative effects on *C. radiatus* caused by the short period outside the culture room could be observed. In a single-well counting pretest, the recognition of live cells and EV-producing cells was done with five independent observers (Yun 31 (live)/10 (EV producers), Vera 29/11, Fatemeh 30/9, Mona 31/9 and Mimi 30/10). Time-lapse video capture was carried out using the same microscope system in the auto-exposure mode. The videos were generated from the time-lapse pictures and edited using the software ImageJ v.1.53c. The size distribution of EVs was determined by microscopy from an old culture. The diameters of all vesicles in the field of vision were determined using the ImageJ software. The fate of the vesicles was determined by evaluation of nine video recordings (among them Supplementary Video 1). Thirty-one EVs that were in contact with a neighbouring algal cell were observed over up to 18 h.

### Single-cell tracking

Six independent cultures were grown to prepare old cells. The old cells were then purified to minimize bacteria following the process described above. The cultures were diluted to a density of 8 cells per ml and then 100 μl was added to each well of a 96-well plate. Individual cells were tracked under the microscope every day for 1 week except day 4. The final fate (rejuvenation or non-rejuvenation) of the cells producing EV was recorded.

### cLSM observation of *C. radiatus*

The fluorescence probes, 5(6)-carboxy-2’-7’dichlorofluorescin diacetate (CDCFDA) (Sigma-Aldrich) and Nile Red (Cayman Chemical) were used for detection of ROS, autophagy and lipids in *C. radiatus* cells and extracellular vesicles. The stock solution of CDCFDA was prepared using dimethylsulfoxide at concentrations of 24.7 mM and 100 mM. Nile Red was dissolved in acetone at a concentration of 0.3 mM. The final concentration was 5 μM for CDCFDA and Nile Red. Cells and vesicles were stained by each dye for at least 15 min in the dark. *C. radiatus* micrographs were acquired using a cLSM 880 microscope (Zeiss) equipped with a ×20/0.8 Plan Apochromat objective (Zeiss) at a resolution of 1,024 × 1,024 pixels with a pixel scaling of 210 × 210 nm, zoom factor 2 and 12 bit depth. CDCFDA, Nile Red, as well as chlorophyll autofluorescence were excited with 25% transmission of a 405 nm laser diode and emissions were recorded via an main beam splitter (MBS) 405, with a pinhole of 1.04 AU, 1.03 µs pixel dwell time and 4 times averaging of unidirectional scans. Spectral windows for the detection channels were defined as 480–540 nm for CDCFDA, 554–638 nm for polar lipids of Nile Red staining, 515–585 nm for neutral lipids of Nile Red staining, as well as 670–740 nm for the chlorophyll autofluorescence running at a detector gain of 500–525 nm. The bright-field signal was acquired as the transmitted light of the 405 nm laser diode recorded by a transmitted light photo multiplier tube (T-PMT) at a gain of 200.

### Quantification of ROS production induced by the bacterial strain CS1

CS1 in MB culture was collected by centrifugation at 13,200 *g* for 5 min and adjusted to OD_600_ = 0.2 with f/2+Si medium. Old *C. radiatus* cells were treated with dense (OD_600_ = 0.2) CS1 in f/2+Si medium at a ratio of 1:1 for 24 h. Control cultures were maintained in parallel. CDCFDA dye at 5 µM final concentration was then added to the culture. After washing with dye-free f/2+Si medium three times, the fluorescence of cells and EVs was recorded using a Varioskan Flash multimode reader (Thermo Fisher) with the following parameters: the black solid 96-well plate was shaken at a speed of 300 r.p.m. and diameter of 5 mm for 20 s and then stopped for 50 s before measurement. For CDCFDA fluorescence detection, the excitation wavelength was 488 nm with a bandwidth of 12 nm, and the emission wavelength was set to 520 nm. The acquisition mode was set as ‘read from plate top’. The measurement time was 100 ms. Multipoint mode was used with a safety zone of 1.4 mm.

To prepare the bacterial culture filtrates for metabolomics experiments, bacteria were transferred in f/2+Si medium supplemented with the amino acids glycine, glutamic acid, threonine, tyrosine, serine, leucine, isoleucine and valine (each at 0.3 mM final concentration) and grown to an OD_600_ of 0.1 (after ~2 days at 28 °C with 150 r.p.m. shaking). The bacteria were removed by centrifugation at 16,100 *g* for 1 min and subsequent filtration through 0.2 µm filters. The young and old *C. radiatus* cells were inoculated into these bacterial filtrates. The f/2+Si+amino acids medium was used as a control. For metabolomics experiments, incubations were run for 3 days. In a pre-experiment, it was verified that the amino acids did not affect algal growth and EV production.

### Metabolomics analysis

For untargeted metabolomics profiling of algal intra- and extracellular metabolites, 20 ml of cultures were transferred to Falcon tubes (50 ml) and centrifuged at 1,200 *g* for 15 min. Approximately 12 ml of the supernatant containing dissolved metabolites as well as the vesicles were carefully transferred to new Falcon tubes. The remaining cultures were centrifuged again at 1,200 *g* for 5 min and the remaining supernatant was pipetted off and combined with the first fraction. The combined supernatant was loaded on a solid-phase extraction cartridge (SPE, Oasis PRiME HLB 3 cc column, Waters) and eluted according to manufacturer protocol.

The cell pellet was resuspended with 3 ml methanol (Sigma-Aldrich) and the suspension was sonicated using a Bandelin Sonopuls HD 2070 ultrasonic homogenizer for 20 s. The resulting lysate was centrifuged for 20 min at 4,000 *g* to remove cell debris. The resulting methanolic extracts were transferred into new glass vials. After dilution with water, the supernatant was loaded on an SPE cartridge (Oasis PRiME HLB 3 cc column, Waters) for extraction following manufacturer recommendations. All samples were dried in a Vacufuge plus vacuum concentrator (Eppendorf) overnight. The dried samples were dissolved in 300 µl methanol and transferred into 1.5 ml Eppendorf tubes for centrifugation at 16,100 *g* for 10 min. The supernatant was transferred into the insert of a glass vial and submitted for MS analysis. Volumes of 5 µl of each sample were combined to obtain the quality control (QC) pools for endometabolites and exometabolites. A 1 µl sample was injected into the UHPLC–HR–MS (UltiMate 3000 UHPLC Dionex) equipped with an Accucore C18 column (100 × 2.1 mm, 2.6 µm, Thermo Fisher) coupled to a Q-Exactive Plus Orbitrap mass spectrometer (Thermo Fisher). The LC separation was performed starting with 100% of A: aqueous phase (2% acetonitrile, 0.1% formic acid in water) and increasing with B: acetonitrile phase (100% acetonitrile) from 0.2 to 8 min until reaching 100% B. This was held for 3 min before switching back to 100% of the water phase and equilibration for 1 min. The flow rate was 0.4 ml min^−1^. The column oven was controlled at 25.0 °C. Mass spectrometry was conducted in positive and negative modes with a scan range of *m*/*z* 80–1,200 at a peak resolution of 70,000, and heated electrospray ionization was used for ionization. AGC target was set to 3 × 106 and maximum ion time was set to 200 ms. The MS/MS spectra of precursor ions, selected with an inclusion list, were obtained from cell extracts with the above-mentioned parameters and within an isolation window of *m*/*z* 0.4 at a peak resolution of 280,000 (NCE 15, 35, 45). The raw dataset was uploaded and is available at https://www.ebi.ac.uk/metabolights/editor/MTBLS5368/descriptors.

### Quantification of methionine

Samples were prepared as described for the exometabolome samples, but before SPE extraction, isotope labelled ^13^C-methionine (490083, Sigma-Aldrich) dissolved in water was added to reach a final concentration of 20 μM for old cells and and 2 μM for young cells as internal standards. Quantification was performed by integrating peak areas of the labelled and unlabelled methionine pseudomolecular ion.

### Statistical analysis and pathway assignment

Peak picking, deconvolution and tentative identification of the metabolites from raw data were performed using the Compound Discoverer software v.3.3.0.550 (Thermo Fisher). Mass tolerance for MS identification was 5 ppm, retention time tolerance was 0.2 min, minimum MS peak intensity was 1 × 10^5^, and intensity tolerance for isotope search was 50%. Relative standard deviation value was set to 50%. The compound list was exported as a .csv file. The injection sequence of the samples and normalization factors according to cell density are listed in Supplementary Table 2. Statistical analysis and enrichment analysis were performed using MetaboAnalyst 5.0. Principal component analysis was performed to compare overall metabolite pattern similarities among intra- and extracellular extracts. Pairwise comparisons for endometabolomics and exometabolomics profiles were made between (1) old control/young control, (2) old CS1/old control, (3) young CS1/young control, (4) old CS4/old control and (5) young CS4/young control. The differential expression metabolites (*P* < 0.05, FC > 1.5) lists from each pair comparison were exported and used for drawing Venn diagrams using the tool https://bioinformatics.psb.ugent.be/webtools/Venn/. The annotated features were uploaded for enrichment analysis. The identity of selected ions was confirmed by comparison of retention time and MS^2^ spectra between the QC pool and analytical standards.

### Bioassays of microalgal growth and EV production

Bioassays were carried out in 96-well plates. After treatment with antibiotics, young and old algal cells were inoculated with an initial cell density of ~20 cells per well (in 100 µl of f/2+Si medium). The respective compound was added to the culture medium as aqueous solution, and cell counts and EV counts were recorded every other day. The EV-producing cell percentage was calculated as EV-producing cells/total live cells × 100%. IC_50_ values were determined on day 7 or day 8, and EC_50_ values were determined on day 4 by fitting the resulting plots.

### EVs extracts and effects on bacteria

Old cells were used for induction of EV production 2 days after transfer into fresh medium. Diatom cells were removed by filtration on 40 μm filters. The filtrates containing EVs were left to sediment for 30 min for EV enrichment. The supernatant was removed by pipetting to reach an EV density of 2,500–3,000 ml^−1^. EVs were lysed by freezing at −80 °C and thawing. The lysate was filtered through 0.2 µm filters to remove bacteria. The supernatant collected after EV sedimentation from the same preparation was treated identically and served as control. A total of 15 bacterial strains were cultivated as previously described^22^. Bacterial cultures with OD_600_ = 0.6 were collected by centrifugation at room temperature and resuspended in fresh MB medium to reach an OD_600_ = 0.4. For each well, 100 μl of test culture was prepared by combining 50 μl of EV extract or control extract, 1 μl of bacterial inoculum and 49 μl of MB medium in a 96-well plate. Bacterial growth was recorded by measuring OD_600_ using a Varioskan Flash multimode reader (Thermo Fisher) at room temperature for 40 cycles at 30 min per cycle.

### Metabolomics profiling of FACS-purified EVs

The experimental workflow is depicted in Extended Data Fig. 10a. The EV enrichment was performed as described above. The enriched EV samples were stained with the ROS probe CDCFDA for 15 min in the dark before FACS analysis. Vesicles were analysed and sorted with a fluorescence activated cell sorter (BD FACS Aria Fusion (BD Biosciences)). A solution containing 3.5% sea salt (Instant Ocean, Aquarium Systems) was used as FACS running buffer. Sorting temperature was kept at room temperature. Vesicles were sorted into 5 ml FACS tubes (Falcon). Chlorophyll *a* fluorescence was detected in the BV650 channel. The ROS dye CDCFDA was detected in the FITC channel. As single staining for ROS dye CDCFDA could not be created, compensation was not performed. To control for media contamination in the sorted vesicle-containing droplets, Accu-drop beads (BD) were diluted into the corresponding supernatant and sorted as above. Data analysis was performed using BD FACSDiva Software v.8 (BD Biosciences). The total sorted events for each sample are listed in Supplementary Table 3. FACS-purified EV and control samples were dried in a Vacufuge Plus vacuum concentrator (Eppendorf) for 2 h. The dried samples were dissolved in 300 μl of methanol and centrifuged at 16,100 *g* for 10 min. The supernatant of 10 μl was loaded on the LC–MS for untargeted metabolomics profiling as described above. The raw dataset was uploaded to www.ebi.ac.uk/metabolights/MTBLS5401.

### Analysis of EVs for fatty acids and oxylipins using UHPLC–MS/MS

The enriched EV preparation was performed as described above, except that EVs were collected from 2-day-old cells after treatment with 100 μM homocysteine or 10 μM methyl methionine to enhance EV production (Fig. 5b). Cell samples were prepared as described for the metabolomics analysis. All samples were dried in a Vacufuge Plus vacuum concentrator (Eppendorf) overnight. The measurement was based on a previously established protocol for lipidomics analysis^42^. Dried samples were resuspended in 100 µl of methanol and subjected to UHPLC–MS/MS. Fatty acids and oxylipins were analysed with a Nexera X2 UHPLC system (Shimadzu) and a QTRAP 5500 mass spectrometer (AB Sciex) equipped with a Turbo V Source and electrospray ionization. Lipids were separated using an ACQUITY UPLC BEH C18 column (1.7 μm, 2.1 × 100 mm; Waters) at 50 °C with methanol:water:acetic acid ratio of 42.0:58.0:0.01 (v/v/v) at a flow rate of 0.3 ml min^−1^ that was ramped to 80.8:19.2:0.01 (v/v/v) over 11 min and then to 98:2:0.01 (v/v/v) for 5 min. The QTRAP 5500 was operated in negative-ionization mode using scheduled multiple reaction monitoring coupled with information-dependent acquisition. Optimized parameters (collision energy, entrance potential, declustering potential, collision cell exit potential) for fatty acid and oxylipin analysis were adopted, and the curtain gas pressure was set to 35 psi. The retention time and at least 6 diagnostic ions for each compound were confirmed using an external commercially available standard (Cayman Chemical). Quantification was achieved using linear calibration curves for each metabolite.

### Statistics and reproducibility

Sample size was determined on the basis of a previous study that reached a significant result (*P* < 0.05). For example, up to 6 replicates were set for the algal cultivation experiment for nutrients measurement, but 3–4 replicates were set for bacteria–diatom co-cultivation and bioassay experiments following previous experimental results for this diatom strain^22^. For metabolomics experiments, 5 independent biological replicates were used for extraction. For all assays showing error bars, the mean values and standard deviations or standard errors across multiple biological replicates were set as the measures of centre and spread. The number of replicates for each experiment and the type of statistical test used to determine significance are included in respective figure legends. Only statistical comparisons for which *P* < 0.05 are shown in graphs. The graphs, data statistical analysis and fitting for EC_50_ and IC_50_ values were applied using Microsoft Office Excel 2016 and GraphPad Prism v.8.00.

### Reporting summary

Further information on research design is available in the Nature Portfolio Reporting Summary linked to this article.

## Supplementary information

Supplementary Information Supplementary Figs. 1–7 and Tables 1–3.

Reporting Summary

Supplementary Video 1 Video.

Supplementary Video 2 Video.

Supplementary Data 1 Source data.

## Source data

Source Data Fig. 1 Data.

Source Data Fig. 2 Data.

Source Data Fig. 3 Data.

Source Data Fig. 4 Data.

Source Data Fig. 5 Data.

Source Data Extended Data Fig. 1 Data.

Source Data Extended Data Fig. 4 Data.

Source Data Extended Data Fig. 5 Data.

**Extended Data Fig. 6:**
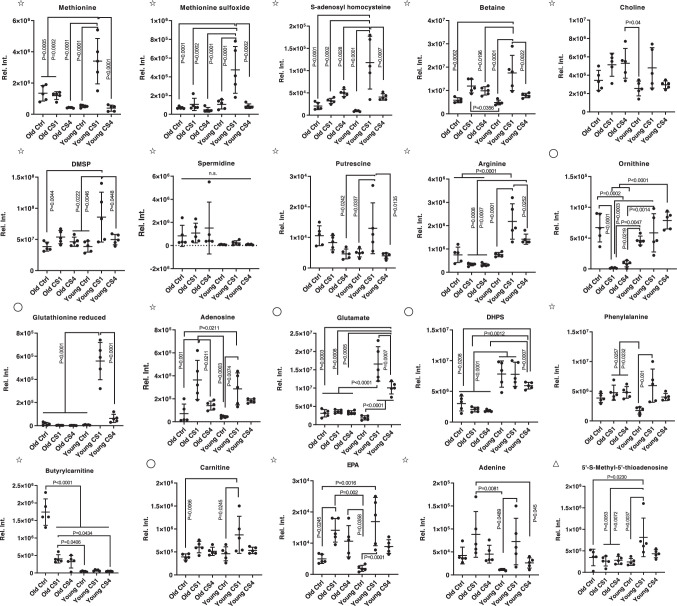
Methionine cycle and lipid endo metabolism. Changes of methionine cycle and lipid metabolism-related endo-metabolites upon treatment of the diatom *C. radiatus* with bacterial filtrates. Significance was assessed by one-way ANOVA Tukey’s multiple comparisons test. Data are presented as mean ± SD based on five independent replicates. ☆ indicates retention time and MS2 spectrum and retention time matched with standards. ○ indicates retention time and MS spectrum matched with standards, Δ concluded by accurate mass. Only statistical comparisons for which P < 0.05 are shown. Source data

Source Data Extended Data Fig. 6 Data.

**Extended Data Fig. 7:**
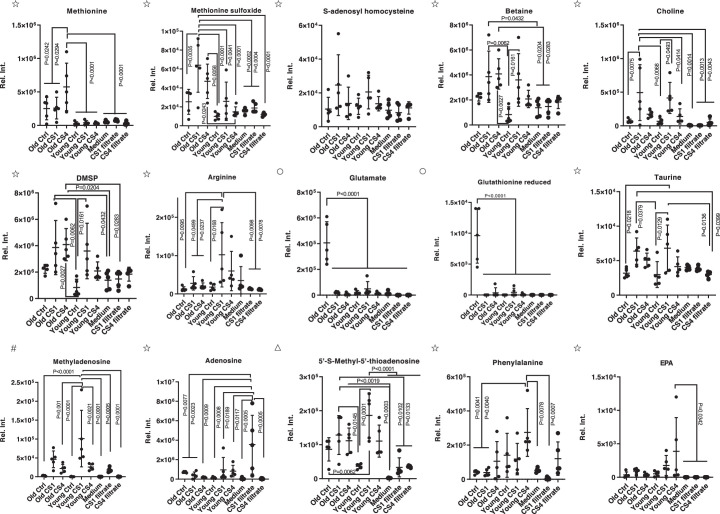
Changes of methionine cycle and lipid exo-metabolism. Changes of methionine cycle and lipid metabolism-related exo-metabolites upon treatment of the diatom *C. radiatus* with bacterial filtrates. Significance was assessed by one-way ANOVA Tukey’s multiple comparisons test. Data are presented as mean ± SD based on five independent replicates. ☆ indicates retention time and MS2 spectrum matched with standards, Δ concluded by accurate mass. ○ indicates retention time and MS spectrum matched with standards. # indicates MS2 spectrum matched with standards, but retention time mismatched suggesting an isomer. Only statistical comparisons for which P < 0.05 are shown. Source data

Source Data Extended Data Fig. 7 Data.

**Extended Data Fig. 8:**
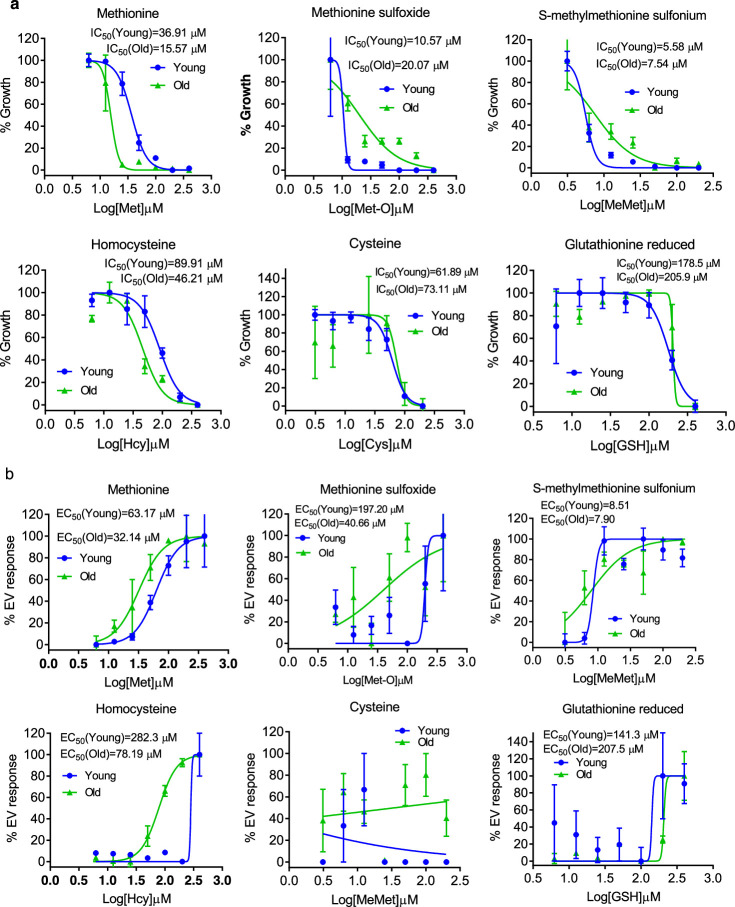
Growth inhibition and EV formation upon stimulation with metabolites from methionine cycle. **a**, Inhibition of growth and **b**, induction of EV formation upon incubation with selected metabolites associated with the methionine cycle. EC_50_ and IC_50_ values are obtained by fitting the data as indicated. EV % data from lag phase day 4 were used. Evaluation of growth inhibition was made from data on the cell density in the stationary phase (day 6, day 7 or day 8). Data are presented as mean ± SD based on three independent replicates. Source data

Source Data Extended Data Fig. 8 Data.

**Extended Data Fig. 9:**
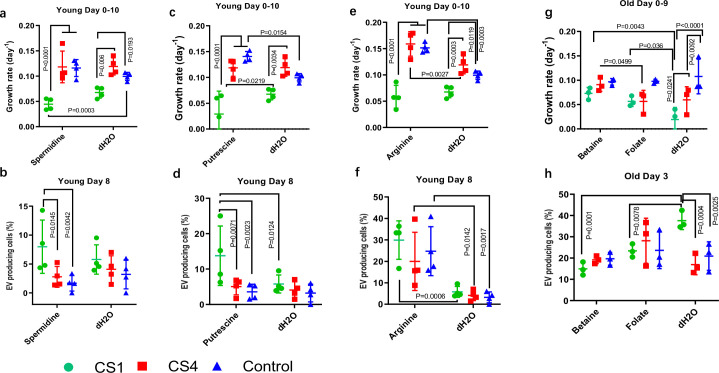
Growth inhibition and EV formation upon stimulation with metabolites from other origin. Treatment of *C. radiatus* with 0.2 mM spermidine, putrescine, arginine, betaine and folate in the presence of bacteria CS1 and CS4 and in controls. The growth rate μ was calculated by the formula, In(N_t_/N_0_)/t with N being the cell numbers at time 0 and t = 10 days. The growth rate **a, c, e** and EV producing cells % **b, d, f** of young cells treated with 0.2 mM spermidine, putrescine and arginine, respectively, in the presence of bacteria CS1 and CS4 (n = 4). The growth rate **g** (day 0–9) and proportion of EV production cells % **h** of old cells was only affected by 0.2 mM betaine and folate in the presence of bacteria CS1 and CS4 (n = 3 independent replicates). The data are presented as mean ± SD. One-way ANOVA with Fisher’s LSD test was used for statistical analysis. Only statistical comparisons for which P < 0.05 are shown. Source data

Source Data Extended Data Fig. 9 Data.

**Extended Data Fig. 10:**
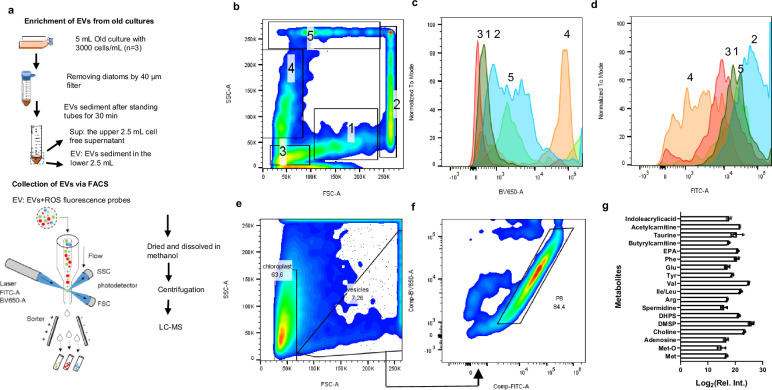
Workflow for FACS-based EV purification and metabolic profiling. **a**, The experimental workflow for FACS-based EV purification and metabolic profiling. Side scatter (SSC) and forward scatter (FSC) referred to two photodetectors that respectively detect the scatter at a ninety-degree angle relative to the laser and along the path of the laser. **b, c**, and **d**, Characterization of particles in the EV sample by FACS. **b**, FSC vs SSC plot. **c**, BV650-A monitors chlorophyll a fluorescence; **d**, FITC-A monitors fluorescence after ROS-sensitive staining. 1 medium-sized vesicles; 2 large vesicles (blue in panels **c** and **d**); 3 small vesicles (red in panels **c** and **d**); 4 chloroplasts (orange in panels **c** and **d**); 5 high granularity particles (light green in panels **c** and **d**). Panels **e**, and **f** indicate the gating strategy used. Supernatant that contained vesicles was pre-gated for events with high FSC-A and low SSC-A. This step excludes SSC-high/FSC-low chloroplasts. Vesicles were then defined as ROS-positive with a low Chlorophyll-A fluorescence.**, f**, Sorting of EVs by FACS. BV650-A vs FITC-A plot. P8 indicates a defined region that was characterized as vesicles and was selected for the collection of EVs for further analysis. The supernatant with equal events of standard beads was collected as a control. **g**, The metabolites detection of FACS purified EVs from old cells by LC-MS. Relative abundances were calculated by subtracting the response of EV sample from blank control and transforming via Log_2_. Error bars are +/- SD of mean from independent triplicates. Source data

Source Data Extended Data Fig. 10 Data.

## Data Availability

The metabolomics datasets MTBLS5368 and MTBLS5401 were approved by the Metabolights curation team and are respectively available at https://www.ebi.ac.uk/metabolights/reviewer3a314956-694c-4080-8495-1c824f20c423 and https://www.ebi.ac.uk/metabolights/reviewerbead6125-d665-4582-8e70-89076285d592. Microscopic data (pictures and movies) are deposited in figshare at 10.6084/m9.figshare.25425154.v1 (ref. ^43^). *Coscinodiscus radiatus* was obtained from the Roscoff culture collection RCC 7277. The two bacterial strains used in this study are available from the corresponding author upon reasonable request. All other datasets generated and/or analysed during the current study are provided as Source data.
