# Supplementary material for: Bacteria modulate microalgal aging physiology through the induction of extracellular vesicle production to remove harmful metabolites
Source: Nat Microbiol. 2024 Aug 14;9(9):2356–68. doi: 10.1038/s41564-024-01746-2 (PMC11371645; doi:10.1038/s41564-024-01746-2)
Supplement: Supplementary file 1 — Supplementary Figs. 1–7 and Tables 1–3. [file 41564_2024_1746_MOESM1_ESM.pdf]

nature microbiology

Supplementary information https://doi.org/10.1038/s41564-024-01746-2

Bacteria modulate microalgal aging physiology through the induction of extracellular vesicle production to remove harmful metabolites

In the format provided by the authors and unedited

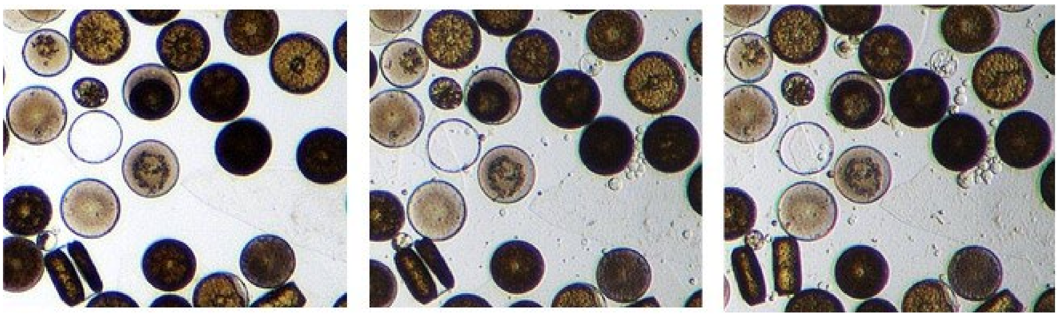

Supplementary Information video 1. Time-lapse capture for old cells EV production in *C. radiatus*. The images were automatically captured every 20 minutes per frame for 18 h 20 min (see linked file).

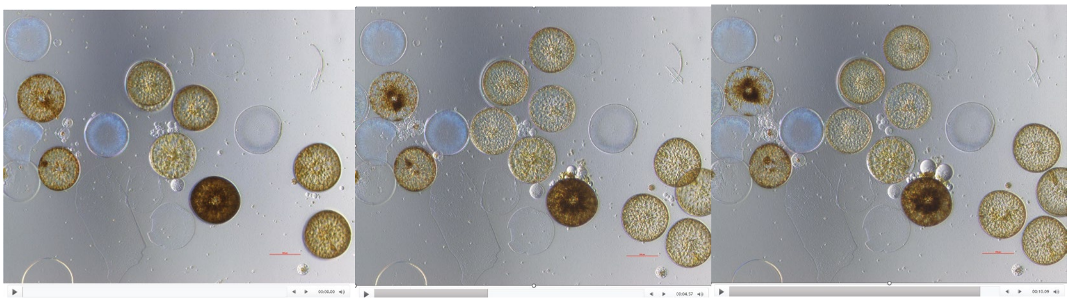

Supplementary Information Video 2 14 h time-lapse capture observing *C. radiatus* EV production after treatment with 50 µM methionine (see linked file).

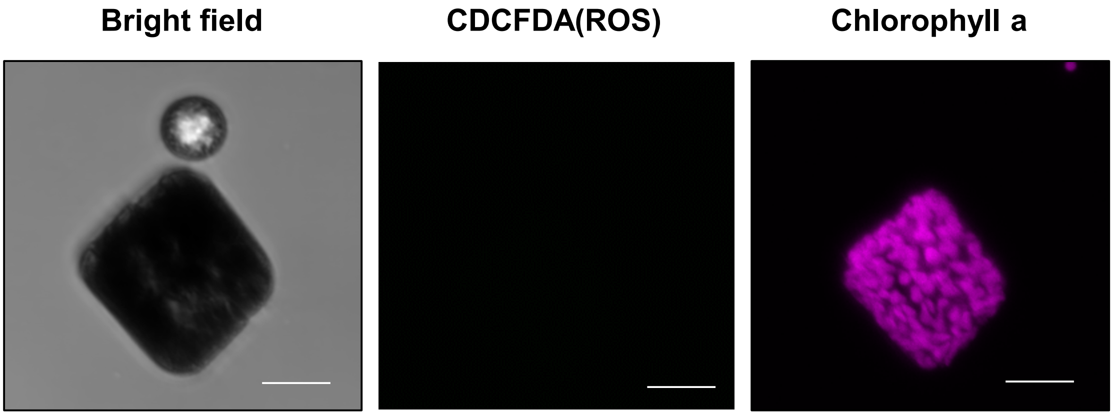

Supplementary Information Fig. 1 Non-stained cell as an example of a negative control for quantification of CDCFDA (ROS) signal intensity in the EV and the producer cell after inoculating in fresh medium. Image of a single EV producing cell from cLSM 3D scanning. The average intensity of all z-axis stacks is presented. The brightness of ROS fluorescence was adjusted to the same level as chlorophyll a. Scale bar=20 μm. The images with these results are observed at least three times.

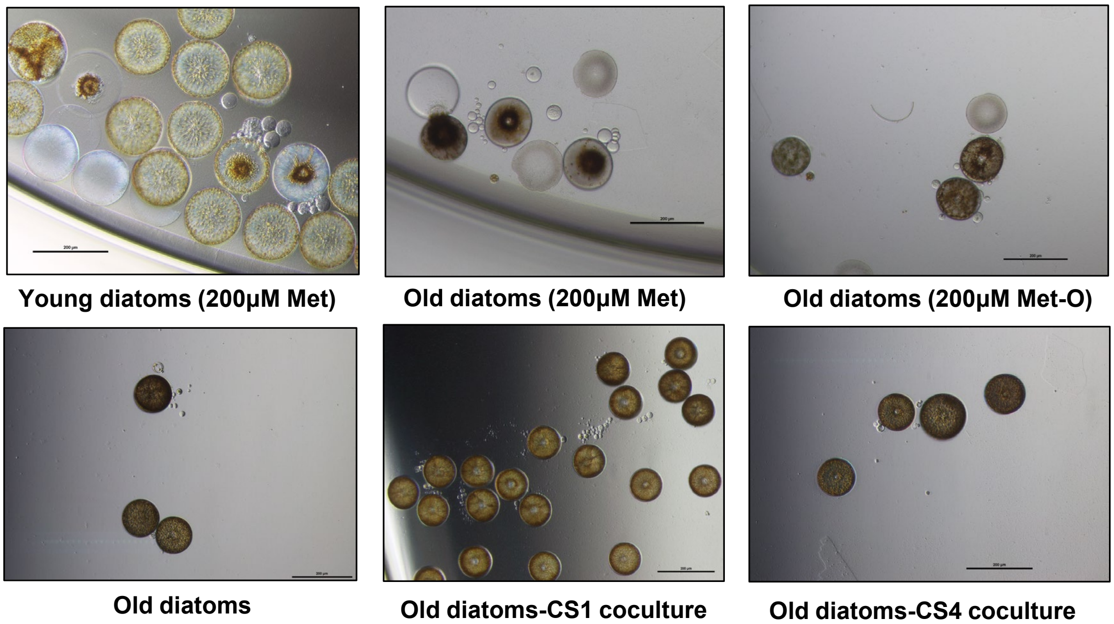

Supplementary Information Fig. 2 PCD like cellular morphology accompanied by EV production was only observed in treatments with high concentrations of methionine (Met) and methionine sulfoxide (Met-O) (upper panel). No PCD-like cellular morphology can be seen from EV-producing old diatoms alone or with the bacteria CS1 and CS4. Scale bars=200 µm. Images with similar results were typical and can be observed repeatedly.

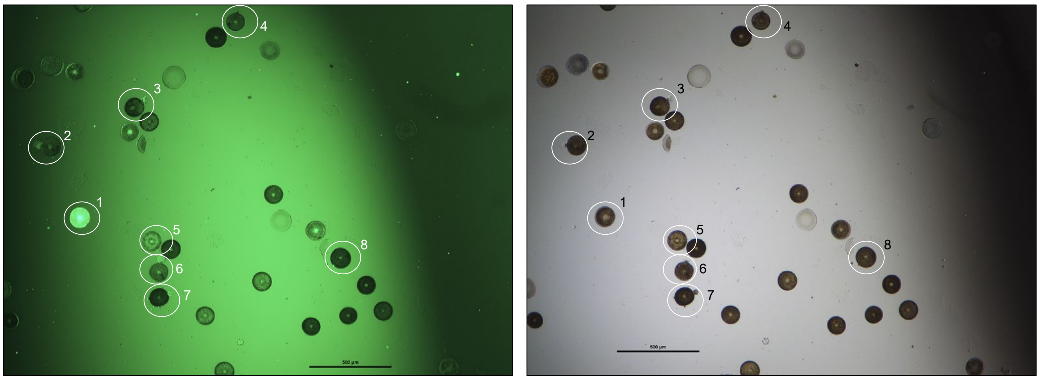

Supplementary Information Fig. 3 Fluorescence microscopic observation of old diatom cells after ROS probe (CDCFDA) staining. The cells were inoculated in fresh medium for 2 days. The left panel showed ROS channel, and the right the bright field observation. The cell marked with the cycle 1 indicates a ROS-positive cell. The cells marked with cycles 2-7 indicate EV-producing cells that did not show obvious ROS signals in cells. Scale bar=500 μm. Images with similar results were observed at least twice.

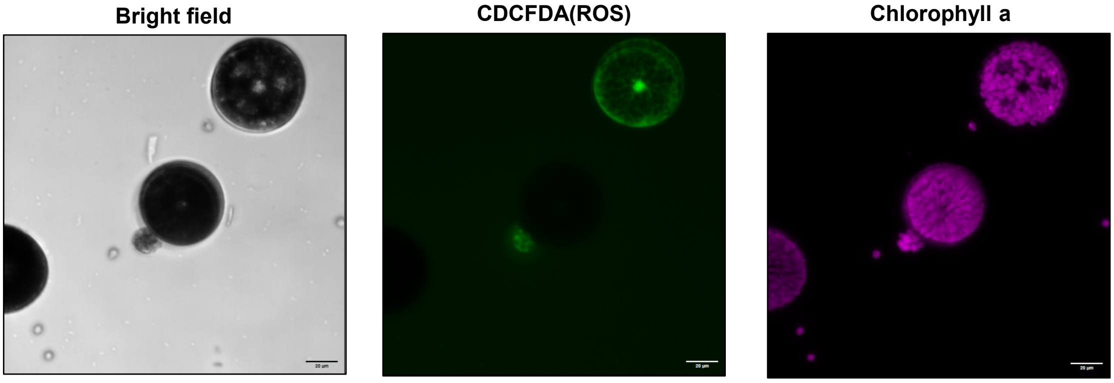

Supplementary Information Fig. 4 cLSM observation of a ROS-positive cell and an EV-producing cell stained by the ROS probe CDCFDA. Diatom cells were prepared after inoculating in fresh medium for 1 day. Image of a single EV-producing cell from cLSM 3D scanning. The average intensity of all z-axis stacks of 21 slices is presented. Scale bar=20 μm. Images with similar results were observed at least twice.

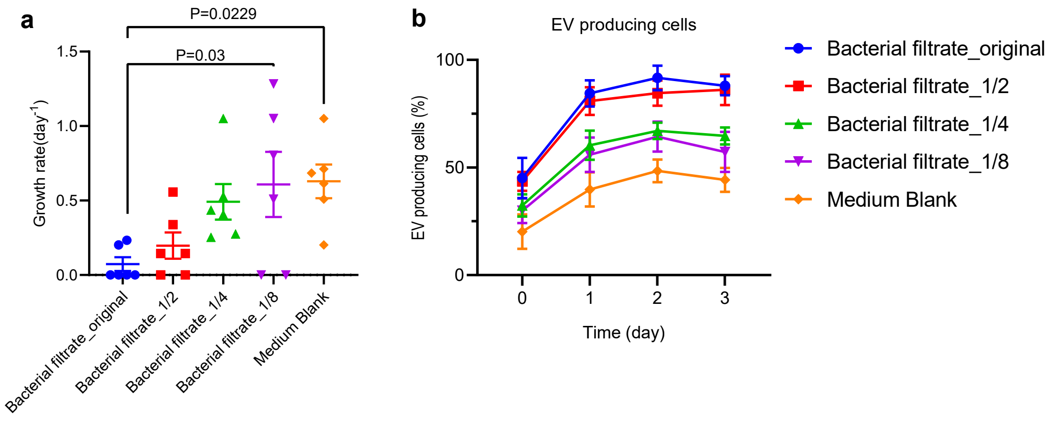

Supplementary Information Fig. 5 Dose-dependent growth and EV production in response to bacterial filtrates. The dilutions 1/2, 1/4, and 1/8 of the 0.2 µm filtrate derived from a culture of the bacterium CS1 grown in f/2+Si medium supplemented with the amino acids, glycine, glutamic acid, threonine, tyrosine, serine, leucine, isoleucine, and valine (each 0.3 mM final concentration) with an OD600 of 0.097 were applied to treat old *C. radiatus*. The tests were carried out in a final volume of 220 µL/well in a 96-well plate. *C. radiatus* cell counts **a**, and the proportions of EV-producing cells **b**, were recorded during 3 days. Data expressed as mean ±SE from six replicates. Statistical analysis was performed by one-way ANOVA using Sidak's multiple comparisons test. Only statistical comparisons for which $P<0.05$ are shown.

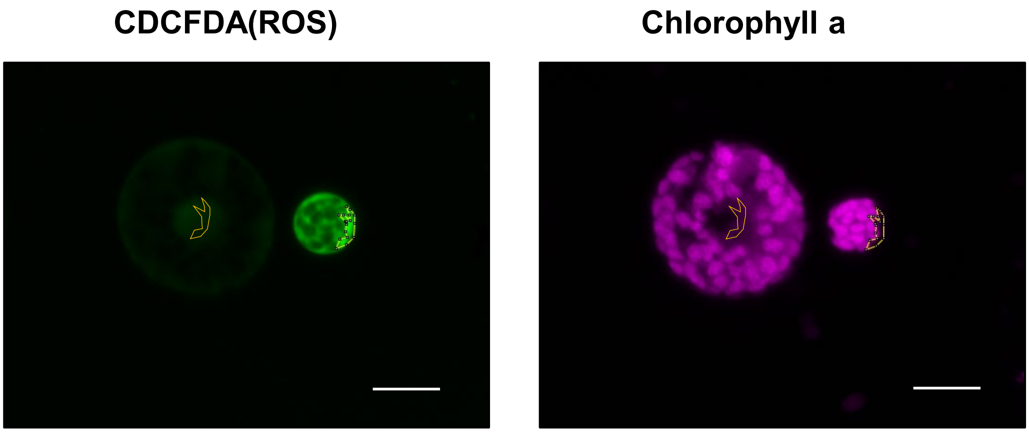

Supplementary Information Fig. 6 An example is shown how the region is selected to exclude chloroplasts to define regions of interest (ROI) for analysis of the mean fluorescent intensity of the ROS probe CDCFDA. Scale bar=20 μm.

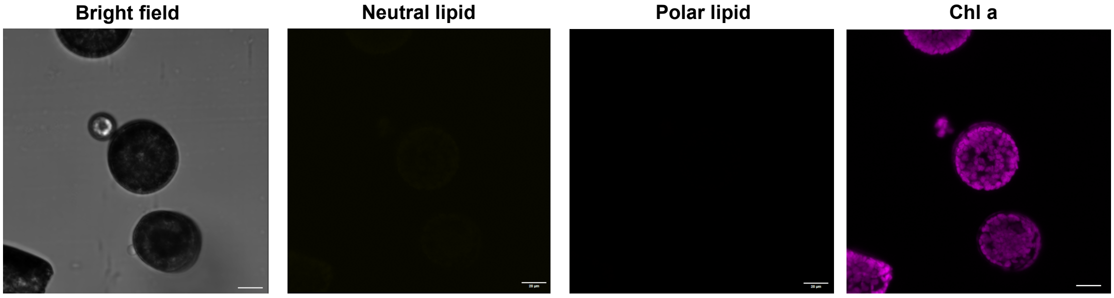

Supplementary Information Fig. 7 Non-stained cells were set as negative control for quantification of Nile red (neutral and polar lipids) signal intensity in the EV and the producer cell. The brightness of Nile red was adjusted to the same level as chlorophyll a. Scale bar=20 μm. Images with these results are observed at least three times.

Supplementary Information Table 1. Results of bioassays on *C. radiatus* growth inhibition (IC_{50}) and EV production (EC_{50})

Metabolite	EC_{50} for Old/Young cells (μM)	IC_{50} for Old/Young cells (μM)
Met	32.14/63.17	15.57/36.91
Hcy	78.19/282.3	46.21/89.91
Cys	>400/>400	73.11/
GSH	207.5/141.3	205.9/178.5
Spermidine	>400/>400	>400/>400
Arg	>400/>400	>400/>400
Betaine	*	*
Folate	*	*
Met-O	40.66/197.2	20.07/10.57
DMSP	*	*
DMSOP	*	*
Adenosine	*	*

*refers to no apparent EV inducing or growth inhibition effects can be observed from a single concentration of 400 μM tests.

Supplementary Information Table 2. Cell counts/ 100 μL after 3 days incubation from metabolomics samples.

	Old cells/100 μL			Young cells/100 μL		
Reps	Control	CS1	CS4	Control	CS1	CS4
I	49	28	37	83	17	26
II	56	22	41	63	37	49
III	70	37	32	57	22	42
IV	65	26	51	57	46	48
V	67	36	45	89	11	33

Supplementary Information Table 3. Events from EV sample and the beads counts from control by FACS

Events	Rep1	Rep2	Rep3
EV(+CDCFDA)	12000	18000	14483
Control (supernatant with beads)	15000	15000	15000
