# Supplementary material for: Bacteria modulate microalgal aging physiology through the induction of extracellular vesicle production to remove harmful metabolites
Source: Nat Microbiol. 2024 Aug 14;9(9):2356–68. doi: 10.1038/s41564-024-01746-2 (PMC11371645; doi:10.1038/s41564-024-01746-2)
Supplement: Supplementary file 2 — Reporting Summary [file 41564_2024_1746_MOESM2_ESM.pdf]

nature portfolio

Corresponding author(s): Georg Pohnert

Last updated by author(s): May 7, 2024

Reporting Summary

Nature Portfolio wishes to improve the reproducibility of the work that we publish. This form provides structure for consistency and transparency in reporting. For further information on Nature Portfolio policies, see our Editorial Policies and the Editorial Policy Checklist.

Statistics

For all statistical analyses, confirm that the following items are present in the figure legend, table legend, main text, or Methods section.

n/a	Confirmed	
☐	☒	The exact sample size (*n*) for each experimental group/condition, given as a discrete number and unit of measurement
☐	☒	A statement on whether measurements were taken from distinct samples or whether the same sample was measured repeatedly
☐	☒	The statistical test(s) used AND whether they are one- or two-sided<br>*Only common tests should be described solely by name; describe more complex techniques in the Methods section.*
☒	☐	A description of all covariates tested
☐	☒	A description of any assumptions or corrections, such as tests of normality and adjustment for multiple comparisons
☐	☒	A full description of the statistical parameters including central tendency (e.g. means) or other basic estimates (e.g. regression coefficient) AND variation (e.g. standard deviation) or associated estimates of uncertainty (e.g. confidence intervals)
☐	☒	For null hypothesis testing, the test statistic (e.g. *F*, *t*, *r*) with confidence intervals, effect sizes, degrees of freedom and *P* value noted<br>*Give P values as exact values whenever suitable.*
☒	☐	For Bayesian analysis, information on the choice of priors and Markov chain Monte Carlo settings
☒	☐	For hierarchical and complex designs, identification of the appropriate level for tests and full reporting of outcomes
☒	☐	Estimates of effect sizes (e.g. Cohen's *d*, Pearson's *r*), indicating how they were calculated

Our web collection on statistics for biologists contains articles on many of the points above.

Software and code

Policy information about availability of computer code

Data collection	The cell counting, EV cell identification, and time-lapse capture was performed via light microscope Leica DM2500 (Leica, Wetzlar, Germany) equipped with a CCD system.<br>The metabolomics data were collected by UltiMate 3000 UHPLC Dionex-Q-Exactive Plus Orbitrap (Thermo Fisher Scientific).<br>The cLSM micrographs were acquired using a cLSM 880 microscope (Zeiss, Oberkochen, Germany).<br>The fluorescence of stained cells and EVs was recorded using a Varioskan Flash Multimode Reader (Thermo Fisher Scientific, MA, USA).<br>Vesicles were analyzed and sorted with a BD FACSAriaTM Fusion (BD Biosciences , San Jose, CA, USA).<br>Fatty acids and oxylipins were analyzed with a Nexera X2 UHPLC system (Shimadzu Corporation, Kyoto, Japan) and a QTRAP 5500 mass spectrometer (AB Sciex, ON L4K 4V8, Canada).<br>FACS was performed by BD FACSAria™ Fusion (BD Biosciences , San Jose, CA, USA)
Data analysis	The Nikon software NIC-elements D 4.30.00 was used for the photographic documentation.<br>The time-lapse pictures were edited by the software ImageJ version 1.53c.<br>The fluorescence images were analysis by the software ImageJ version 1.53c.<br>The metabolomics data were analyzed by Compound Discoverer™ software version 3.3.0.550 (Thermo Fisher Scientific) and on-line software MetaboAnalyst 5.0.<br>Venn diagrams using the tool https://bioinformatics.psb.ugent.be/webtools/Venn/.<br>The graphs, data statistical analysis and fitting for EC50 and IC50 values were applied by the software Microsoft office Excel 2016 and GraphPad Prism version 8.00.<br>FACS data was analyzed by BD FACSDiva™ Software v8(BD Biosciences , San Jose, CA, USA)

For manuscripts utilizing custom algorithms or software that are central to the research but not yet described in published literature, software must be made available to editors and reviewers. We strongly encourage code deposition in a community repository (e.g. GitHub). See the Nature Portfolio guidelines for submitting code & software for further information.

Data

Policy information about availability of data

All manuscripts must include a data availability statement. This statement should provide the following information, where applicable:

- Accession codes, unique identifiers, or web links for publicly available datasets
- A description of any restrictions on data availability
- For clinical datasets or third party data, please ensure that the statement adheres to our policy

The metabolomics datasets MTBLS5368 and MTBLS5401 are approved by the Metabolights curation team and deposited at Metabolights. Microscopic data (pictures and movies) are deposited in figshare.com under the accession number 10.6084/m9.figshare.25425154.Two bacteria strains used in this study are accessible via contacting corresponding author. All other datasets generated during and/or analysed during the current study are provided as Source Data files.

Human research participants

Policy information about studies involving human research participants and Sex and Gender in Research.

Reporting on sex and gender	not applicable
Population characteristics	not applicable
Recruitment	not applicable
Ethics oversight	not applicable

Note that full information on the approval of the study protocol must also be provided in the manuscript.

Field-specific reporting

Please select the one below that is the best fit for your research. If you are not sure, read the appropriate sections before making your selection.

☒ Life sciences ☐ Behavioural & social sciences ☐ Ecological, evolutionary & environmental sciences

For a reference copy of the document with all sections, see nature.com/documents/nr-reporting-summary-flat.pdf

Life sciences study design

All studies must disclose on these points even when the disclosure is negative.

Sample size	Sample size was determined based on previous study that can reach a significant result (P<0.05) and considering the technical parameters as well. For example, up to six replicates were set for algal cultivation experiment for nutrients measurement. But 3-4 replicates were set for bacteria-diatom co-cultivation, and bioassay experiments due to the previous experiment results for the growth of this diatom strain (see reference aem.01619-22). For metabolomics experiment, five independent biological replicates were set for extraction according to previous experiment results in the lab(see reference 10.1016/j.phytochem.2022.113267).
Data exclusions	No data were excluded from analysis.
Replication	All experimental findings were reproducible and all experiments included at least three biological replications.
Randomization	For measurements involved in sampling, the flasks with experimental cultures were fully mixed before taking samples to avoid to bring variations. For cultivation and bioassay experiments using cell counting under microscope, all cells per well were counted by adjusting initial cell density for each well to a proper level, but not partial cells of a selected view field in a well were counted. For metabolomics, quality controls and blanks were applied and checked to ensure no obvious time-dependent fluctuations existed from instrument, thus no randomization was used.
Blinding	For cLSM imaging and lipid analysis, the persons who analyzed the samples were unaware of the sample identity.<br>Blinding was not possible for cell counting experiments as the experimental arrangement is complicated to randomize (multichannel pipette is necessary for sample preparing) and the same person set the experiment arrangement and measured the results.

Reporting for specific materials, systems and methods

We require information from authors about some types of materials, experimental systems and methods used in many studies. Here, indicate whether each material, system or method listed is relevant to your study. If you are not sure if a list item applies to your research, read the appropriate section before selecting a response.

Materials & experimental systems

n/a	Involved in the study	
☒	☐	Antibodies
☐	☒	Eukaryotic cell lines
☒	☐	Palaeontology and archaeology
☒	☐	Animals and other organisms
☒	☐	Clinical data
☒	☐	Dual use research of concern

Methods

n/a	Involved in the study	
☒	☐	ChIP-seq
☐	☒	Flow cytometry
☒	☐	MRI-based neuroimaging

Eukaryotic cell lines

Policy information about cell lines and Sex and Gender in Research

Cell line source(s)	Algal strain Coscinodiscus radiatus RCC 7277 was commercially available from Roscoff culture collection (https://roscoff-culture-collection.org/rcc-strain-details/7277). Bacterial strains can be obtained upon request from the correspoinding author.
Authentication	None of the cell lines are authenticated.
Mycoplasma contamination	The cell line was not tested for Mycoplasma contamination, but no contamination was observed.
Commonly misidentified lines (See ICLAC register)	Not relevant for marine single-cell alga.

Flow Cytometry

Plots

Confirm that:

☒ The axis labels state the marker and fluorochrome used (e.g. CD4-FITC).

☒ The axis scales are clearly visible. Include numbers along axes only for bottom left plot of group (a 'group' is an analysis of identical markers).

☒ All plots are contour plots with outliers or pseudocolor plots.

☒ A numerical value for number of cells or percentage (with statistics) is provided.

Methodology

Sample preparation	The filtrates containing EVs were left to sediment for 30 min for EVs enrichment. The enriched EV samples were stained with the ROS probe 5(6)-carboxy-2’-7’dichlorofluorescin diacetate for 15 min in the dark before FACS analysis. The running buffer contains 3.5% sea-salt (Instant Ocean, Aquarium Systems, Mentor, OH, US).
Instrument	BD FACSAria™ Fusion (BD Biosciences , San Jose, CA, USA)
Software	BD FACSDiva™ Software (BD Biosciences , San Jose, CA, USA)
Cell population abundance	For the identified vesicle population P8, in three replicates, 12000, 18000, and 14483 events were sorted for further LC-MS analysis.
Gating strategy	For collecting purified vesicles, firstly, chloroplasts were identified by plotting the autofluorescence of chlorophyll BV650-A, and none-ROS staining (FITC-A). Therefore, a threshold was applied on the FSC-A to exclude relative small size chloroplasts. Then, a relative high ROS staining (FITC-A) population, P8, in BV650-A vs FITC-A plot was selected for sorting vesicles. This gating strategy is showed in Extended Data Fig 8.

☒ Tick this box to confirm that a figure exemplifying the gating strategy is provided in the Supplementary Information.
